# Molecular Characterization of UGT94F2 and UGT86C4, Two Glycosyltransferases from *Picrorhiza kurrooa*: Comparative Structural Insight and Evaluation of Substrate Recognition

**DOI:** 10.1371/journal.pone.0073804

**Published:** 2013-09-16

**Authors:** Wajid Waheed Bhat, Niha Dhar, Sumeer Razdan, Satiander Rana, Rukmankesh Mehra, Amit Nargotra, Rekha S. Dhar, Nasheeman Ashraf, Ram Vishwakarma, Surrinder K. Lattoo

**Affiliations:** 1 Plant Biotechnology, Indian Institute of Integrative Medicine (CSIR), Jammu, India; 2 Discovery Informatics, Indian Institute of Integrative Medicine (CSIR), Jammu, India; 3 Medicinal Chemistry, Indian Institute of Integrative Medicine (CSIR), Canal Road, Jammu, India; University of Waikato, New Zealand

## Abstract

Uridine diphosphate glycosyltransferases (UGTs) are pivotal in the process of glycosylation for decorating natural products with sugars. It is one of the versatile mechanisms in determining chemical complexity and diversity for the production of suite of pharmacologically active plant natural products. *Picrorhiza kurrooa* is a highly reputed medicinal herb known for its hepato-protective properties which are attributed to a novel group of iridoid glycosides known as picrosides. Although the plant is well studied in terms of its pharmacological properties, very little is known about the biosynthesis of these important secondary metabolites. In this study, we identified two family-1 glucosyltransferases from *P. kurrooa*. The full length cDNAs of UGT94F4 and UGT86C4 contained open reading frames of 1455 and 1422 nucleotides, encoding polypeptides of 484 and 473 amino acids respectively. UGT94F2 and UGT86C4 showed differential expression pattern in leaves, rhizomes and inflorescence. To elucidate whether the differential expression pattern of the two *Picrorhiza* UGTs correlate with transcriptional regulation *via* their promoters and to identify elements that could be recognized by known iridoid-specific transcription factors, upstream regions of each gene were isolated and scanned for putative *cis*-regulatory elements. Interestingly, the presence of *cis*-regulatory elements within the promoter regions of each gene correlated positively with their expression profiles in response to different phytohormones. HPLC analysis of picrosides extracted from different tissues and elicitor-treated samples showed a significant increase in picroside levels, corroborating well with the expression profile of UGT94F2 possibly indicating its implication in picroside biosynthesis. Using homology modeling and molecular docking studies, we provide an insight into the donor and acceptor specificities of both UGTs identified in this study. UGT94F2 was predicted to be an iridoid-specific glucosyltransferase having maximum binding affinity towards 7-deoxyloganetin while as UGT86C4 was predicted to be a kaempferol-specific glucosyltransferase. These are the first UGTs being reported from *P. kurrooa.*

## Introduction

Glycosylation is a common modification reaction in plant metabolism and is invariably associated with the production of large array of secondary metabolites. The enzymes that lead to glycoside formation are known as uridine diphosphate glycosyltransferases (UGTs), members of family-1 glycosyltransferases superfamily. It contains over 80 families of enzymes [1], [2] and typically accomplish this task by transferring a UDP-activated glucose to a corresponding acceptor molecule. UGTs utilize UDP-activated sugars as donors and transfer their sugar moiety to various acceptors. Plant family-1 UDP-dependent glycosyltransferases catalyse the glycosylation of a plethora of bioactive natural products and is often the final step in the biosynthesis of many plant natural products [3], enhancing their solubility and stability, and facilitating their storage and accumulation in plant cells. Consistent with the variety and complexity of plant natural products, a large number of UGT gene sub-families have evolved for the glycosylation of these molecules [3], [4]. UGTs involved in secondary metabolism share a conserved 44 amino acid residue motif (60–80% identity) known as the plant secondary product glucosyltransferase box (PSPG). PSPG box has been demonstrated to encompass the UDP–sugar binding moiety [4]–[6]. However, UGTs share relatively low levels of sequence identity, especially within the regions related to acceptor binding, and this feature may be essential to account for the recognition of the huge variety of acceptors and the synthesis of the large number of products.

*Picrorhiza kurrooa* Royle ex. Benth. (Plantaginaceae) is a perennial medicinal herb, also known as ‘*Kutki*’ or ‘*Kadu*’, endemic to north western alpine Himalayas, found at an altitude of 2800 m to 4800 m. It is a reputed herb in the Ayurvedic system of medicine and has been used traditionally to treat liver ailments, dyspepsia, chronic diarrhoea and upper respiratory tract ailments. In modern medicine, it is being used in hepatic disorders, gastric troubles, anaemia, asthma and pregnancy related problems. Most of its pharmacological activities are attributed to novel monoterpene derived iridoid glycosides known as picrosides which include picroside-I, picroside-II and other metabolites like picroside-III, picroside-IV, apocynin, androsin, catechol, kutkoside, etc. [7]. Picroliv, a hepato-protective drug formulation has been prepared from the extracts of *P. kurrooa* in which picroside-I is the major ingredient and therefore, makes this compound a highly valued secondary metabolite of *P. kurrooa*. Studies have shown that picroliv exhibits hepato-protective effect against aflatoxin [8], oxytetracycline [9], carbon tetrachloride [10], paracetamol [11], and alcohol [12], protects against ischemia reperfusion injury of the liver [13] and kidneys [14]. It also exhibits anti-inflammatory [15], immuno-modulatory [16], [17], cardio-protective and neuro-protective [18] and anti-carcinogenic [19], [20] effects.

**Figure 1:**
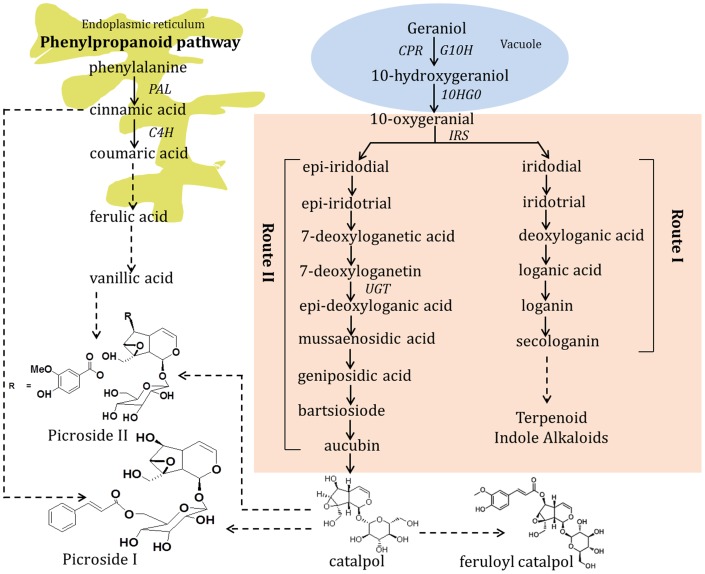
A schematic diagram of the proposed picroside pathway in *Picrorhiza kurrooa.* Picrosides are derived from geranyl diphosphate that can be synthesized both from cytoplasmic MVA and plastidic MEP pathways. CPR: cytochrome P450 reductase; G10H: geraniol 10-hydroxylase; 10 HGO: 10-hydroxygeraniol oxidoreductase; IRS: Iridoid synthase; PAL: phenylalanine ammonia-lyase; C4H: cinnamoyl 4-hydroxylase; Route I: The route leading to the biosynthesis of secologanin, Route II: The route leading to the biosynthesis to picrosides *via* catalpol. Adapted from [62], [63].

Picrosides are essentially iridoid glycosides (cyclopenta-[c]-pyran monoterpenoids), distributed in numerous plant families, usually as glucosides [21]. Iridoids represent bicyclic monoterpenes that possess wide spectrum of pharmacological activities, like anti-inflammatory, anticancer, antibacterial and antifungal activities. There are some iridoids which are used as sex pheromones in certain agriculturally important species of aphids [22]. They are derived from geranyl diphosphate that can be synthesized both from cytoplasmic MVA and plastidic MEP pathways [23]. However, in case of picrosides, non-mevalonate pathway seems to be the main precursor donor [24]. Present knowledge about the biosynthesis of picrosides, and the genes and enzymes involved, is however limited. The prevailing concept is that the precursor iridotrial is cyclized to a limited number of core structures, which are subsequently decorated with functional groups, through various reactions, including oxidation, methylation, and glycosylation as shown in Figure 1. These key steps are considered to be catalysed by two multigene families: 1) cytochrome P450s adding the majority of functional groups, and 2) family-1 glycosyltransferases (UGTs) adding sugars, allowing a vast structural complexity. Few studies have investigated the biochemical and molecular characterization of the picrosides biosynthetic pathway genes [25], [26]. However, the 1-*O*-glucosylation step in the picroside biosynthetic pathway has been poorly characterized and genes for enzymes relevant to the glucosylation step in the picroside biosynthesis are yet to be characterized.

An additional constraint with *P. kurrooa* is that it figures prominently in endangered list of plant species, primarily because of its indiscriminate collection from natural habitats. Owing to its immense therapeutic importance, low content of picrosides and dwindling natural populations, it becomes all the more important to develop elite lines with improved phytochemical content either by classical breeding approaches or through biotechnological interventions for commercial and conservation purposes.

Considerable effort has been focused on understanding the biosynthesis of bioactive picrosides and enhancing their production. Chemosynthesis and manipulation of cell and tissue cultures for picrosides production has also been investigated [27]. However, these methods have not yielded desirable results. One of the most reasonable approaches to enhance the production of picrosides is to employ metabolic engineering. To optimise the production of these natural products, the enzymes involved in their biosynthesis and the methods to regulate the enzyme activity of the rate limiting steps of picroside biosynthesis is imperative. Therefore, the present study was aimed at the identification of glucosyltransferase(s) involved in the glucosylation of picroside precursors. Herein we report the identification of two UDP-glucosyltransferases, designated as UGT86C4 and UGT94F2. Using various bioinformatic approaches including homology modeling and molecular docking studies, we provide an insight into the donor and acceptor specificities of both UGTs identified in the present study. UGT94F2 is apparently responsible for iridoid glucosylation in *P. kurrooa* as 1) it showed maximum binding affinity for iridotrial class of compounds, particularly 7-deoxyloganetin, 2) the expression pattern of UGT94F2 corroborated well with the picroside levels, 3) the binding sites for important transcription factors including MYB, MYC and WRKY, known to be involved in regulating iridoid expression in *Catharunthus roseus,* were found in the *cis*-elements of *Picrorhiza* UGT94F2, and 4) low temperature response elements identified in upstream region of UGT94F2 provided additional support for UGT94F2 to be an iridoid-specific glucosyltransferase, since picroside content has been shown to be higher at low temperatures [24]. The expression pattern of UGT94F2 and UGT86C4 in different tissues of *P. kurrooa* correlated well with picroside abundance and elicitors like methyl jasmonate (MeJA), salicylic acid (SA) and 2, 4-dichlorophenoxyacetic acid (2, 4-D) were found to alter the expression pattern of UGT94F2 and UGT86C4 in a time dependant manner. Furthermore, our results also show possible relationship between specific *cis*-elements identified within promoter sequences of UGT86C4 and UGT94F2, and their corroboration with the expression pattern of UGT86C4 and UGT94F2 in relation to exogenous application of different phytohormones. We anticipate that the present investigation leading to identification of two UGTs may enable to manipulate the production of picrosides in *P. kurrooa* for pharmacological applications.

## Materials and Methods

### Plant Material and Phytohormone Treatment

Cultivated plants of *P. Kurrooa* were originally collected from a private property (Hotel Snowland Resorts), Sonamarg area (Jammu and Kashmir state, India, 34°18′17′′ N, 75°17′8′′ E; 3500 m in altitude) after seeking proper permission from the owner of the property. *P. kurrooa* plants are cultivated therein for aesthetic purposes. *In vitro* cultures were initiated and established from field grown plants at Indian Institute of Integrative Medicine, Srinagar (34°5′24″ N, 74°47′24″ E; 1730 m in altitude) [28]. These were used for the isolation of glucosyltransferases. For SA, MeJA, and 2, 4-D treatments, the plants were mist sprayed over with SA (1 mM), 2, 4-D (50 µM) and MeJA (0.1 mM) solution on both sides of leaves until liquid dripped from the leaves. The plants were covered with transparent polythene. Shoot tips including 4–5 true leaves, from 3–4 plants were excised after 6, 12 and 24 h post treatment and combined as one biological replicate. Fresh tissue samples were immediately frozen in liquid nitrogen and kept at −80°C for further analysis.

### RNA Extraction and cDNA Synthesis

As a first step in identifying glucosyltransferase genes involved in iridoid O-glucosylation in *P. kurrooa,* total RNA was extracted from the leaf tissues using TRIzol reagent following the manufacturer’s instructions (Invitrogen, Carlsbad, CA, USA). Absorbance of RNA samples was determined using a NanoDrop® ND-1000 spectrophotometer (NanoDrop Technologies, Wilmington, DE, USA). RNA quality was assessed by the optical density (OD) ratios at 260 nm/280 nm and by separation on 1% agarose gels followed by visual evaluation of band integrity. Total RNA was treated with DNase I (Fermentas, Burlington, Canada) to eliminate genomic DNA present in the samples. For cDNA synthesis, 2 µg of DNase I treated total RNA was reverse transcribed using Revert-aid premium reverse transcription kit with an modified Adapter-oligo dT primer in a total volume of 20 µL for 1 h at 42°C as described in the instruction manual (Fermentas, Burlington, Canada). The resultant reaction mixture was used as a template for PCR amplification.

### Cloning of UGT86C4 and UGT94F2 from *P. kurrooa*

Cloning of glucosyltransferase cDNAs was performed as described earlier by Nagatoshi et al. [29]. Two degenerate primers, degUGT1F (TSNGTNGCNTAYGTNTSNTTYGG) and degUGT2F (GTNGCNTAYGTNTSNTTYGG) were designed on the basis of amino acid sequences F(L/V)(T/S)HCGWN and CGWNS(T/V)LE respectively in the conserved PSPG box of plant glycosyltransferases. Aliquot of the cDNA was used as a template for PCR amplification in a 20 µl reaction mixture containing 1 µM primer degUGT1F, 0.2 µM 3′ RACE Adapter primer. A portion of the first PCR product was used as the template for nested PCR using the degUGT2F and 3′ RACE adapter primers. The primary and nested PCRs were performed under the following conditions: Denaturation at 94°C for 3 min; 35 cycles at 94°C for 30 s, 45°C for 1 min, and 72°C for 1 min; and a final extension step at 72°C for 10 min. Amplified fragments of 500 bp were recovered from an agarose gel and subcloned into the *pTZ57R/T* cloning vector (Fermentas, Burlington, Canada). Randomly selected cloned inserts were sequenced using a BigDye terminator cycle sequencing kit (Applied Biosystems, Foster City, CA, USA) with an ABI PRISM® 3130XL genetic analyser (Applied Biosystems, Foster City, CA, USA).

The 5′ ends of two putative UGTs were obtained using the SMART™ RACE cDNA amplification kit (Clontech, Palo Alto, CA, USA). The 5′ RACE-ready first-strand cDNA was synthesized following the protocol provided by the manufacturer. For the first PCR amplification of UGT86C4, UGT1-5race-out and UPM (Universal Primer A Mix; Table S1) were used as primers and 5′ RACE-Ready cDNA as the template. For the nested PCR amplification, UGT1-5race-in and NUP (Nested Universal Primer A, Table S1) were used as the nested PCR primers, while the product of the first PCR amplification was used as template. Both the first and the nested PCR amplification procedures were carried out under following conditions: 3 min at 94°C, 32 cycles (35 s at 94°C, 35 s at 60°C, 2 min at 72°C) and 8 min at 72°C. Similarly, the 5′ end of UGT94F2 was amplified using UGT2-5race-out gene specific primer and UPM for first round of PCR followed by a nested PCR using the primary PCR product as template, UGT2-5race-in and NUP as nested primers. Both the primary and the nested PCR amplification procedures were carried out at following condition: 3 min at 94°C, 35 cycles (45 s at 94°C, 35 s at 65°C, 2 min at 72°C) and 8 min at 72°C. The nested amplification products of both 5′ RACE products were purified and cloned into *pTZ57R/T* cloning vector (Fermentas, Burlington, Canada). The ligated DNA was transformed in *E. coli* cells (DH5α; NEB, UK). The clones (25 colonies) were picked individually and grown in 10 mL of Luria-Bertani (LB) medium at 37°C overnight. The plasmid DNA from each clone was extracted using a DNA plasmid Miniprep kit (Promega; Madison, WI, USA) and sequenced using M_13_ primers. DNA sequencing was performed with ABI PRISM 3130XL Genetic Analyser and BigDye Terminator v.3.1 Cycle Sequencing Kit (Applied Biosystems, Foster City, CA, USA).

### Full-length Cloning of UGT94F2 and UGT86C4

By aligning 3′ RACE and 5′ RACE product sequences, the full-length cDNAs of UGT94F2 and UGT86C4 were obtained. The open reading frame (ORF) of both UGT94F2 and UGT86C4 were amplified using single-stranded cDNA template and gene-specific primers (Table S1; PkUGT1Full-F/PkUGT1Full-R and PkUGT2Full-F/PkUGT2Full-R), which cover the start codon and the stop codon regions. A high fidelity proof-reading DNA polymerase (New England Biolabs, Ipswich, MA, USA) was used for amplification under following PCR conditions: One cycle of 98°C for 1 min, 35 cycles of 98°C for 20 s, 60°C for 30 s, 72°C for 2 min. The final extension was at 72°C for 10 min. The resulted amplified full length ORFs were ligated in *pJET* vector (Fermentas, Burlington, Canada) and subcloned into chemically competent *E. coli* cells (DH5α; New England Biolabs, Ipswich, MA, USA). Primers used in the study are listed in Table S1.

### Sequence Analysis of UGT86C4 and UGT96F4

The full length nucleotide sequences obtained were translated using Translate tool (http://www.expasy.ch/tools/dna.html) and the properties of deduced amino acid sequences were estimated using ProtParam (http://www.expasy.ch/tools/protparam.html) and Phobius (http://www.ebi.ac.uk/Tools/pfa/phobius/) programs.

### Phylogenetic Analysis of UGT86C4 and UGT96F4

Protein sequences were retrieved from the GenBank through BLASTp algorithm at the National Centre for Biotechnology Information (NCBI) using UGT94F2and UGT86C4 sequences as query and several UGT orthologs of different families from various plant species were selected. Sequences were aligned using the ClustalW program (http://www.ebi.ac.uk) using default parameters. Neighbour-joining tree was constructed with MEGA 5.10 software [30]. Bootstrap analysis with 1000 replicates was also conducted in order to obtain confidence level for the branches.

### Homology Modeling and Prediction of Three-dimensional Structure of UGT86C4 and UGT94F2

The three-dimensional structure of UGT86C4 and UGT94F2 were performed using PHYRE2 server (**P**rotein **H**omology/analogy **R**ecognition **E**ngine V 2.0) (http://www.sbg.bio.ic.ac.uk/phyre2/html/) using the crystal structure of *Arabidopsis thaliana* UGT72B1 (Protein Data Bank (PDB) Id. 2VCH-A) as template for modeling of both the proteins. Protein refinements of UGT86C4 and UGT94F2 models were performed using Protein Preparation Wizard of the Schrodinger Suite 2012 (http://www.schrodinger.com/). The stereo chemical analysis of the modeled proteins was carried out using Ramachandran plot obtained from Procheck module of the SAVES server (http://services.mbi.ucla.edu/SAVES/). Ligand binding site was predicted using 3DLigandSite (http://www.sbg.bio.ic.ac.uk/3dligandsite/). Structurally, evolutionary, and functionally important regions were identified in deduced protein sequences by ConSurf (http://consurf.tau.ac.il/). Topology of the modeled UGT86C4 and UGT94F2 proteins was analysed using PDBsum (http://www.ebi.ac.uk/thornton-srv/databases/pdbsum/Generate.html).

### Protein Preparation, Identification of Binding Site and Molecular Docking Studies

To gain an understanding of the molecular structure governing acceptor as well as donor specificity of UGT86C4 and UGT94F2, structural alignment of both the proteins was carried out with the crystal structure of UGT78G1 from *Medicago truncatula* (PDB Id. 3HBF). The ligand coordinates at the acceptor site of 3HBF were copied to UGT86C4 and UGT94F2 modeled structures. The grid was generated around the copied ligand at the acceptor sites of UGT86C4 and UGT94F2. The UGT86C4 and UGT94F2 proteins were prepared for docking calculations using the Protein Preparation Workflow (Schrödinger Suite 2012, Protein Preparation Wizard). Ligand docking was carried out for both proteins UGT86C4 and UGT94F2, using Glide XP precision mode. Ligands used for the study included iridotrial, 7-deoxyloganetic acid, 7-deoxyloganetin, apigenin, kaempferol and naringenin.

### Isolation of 5′ Flanking Regions of UGT86C4 and UGT94F2

GenomeWalker DNA libraries were constructed following the user manual provided by the manufacturer (Universal GenomeWalker™ Kit, Clontech, Palo Alto, CA, USA). Genomic DNA of *P. kurrooa* was isolated from fresh leaves using DNeasy Plant mini kit (QIAGEN, Hilden, Germany) according to manufacturer’s protocol with minor modifications [31]. Purified Genomic DNA (3 µg) was digested with *DraI*, *EcoRV*, *PvuI* and *StuI* respectively and recovered using a PCR purification kit (QIAGEN, Hilden, Germany). PCR purified digested DNA were then ligated separately to the AP adaptor (provided in the kit) to construct the genomic walking libraries. For isolation of UGT86C4 gene promoter, the primary PCR was performed using AP1 (provided with the kit) and gwUGT1-out as primers, and constructed libraries as template *via* the following protocol: 5 cycles at 94°C for 25 s and 70°C for 3 min; 30 cycles at 94°C for 25 s, 65°C for 3 min; and at 67°C for 7 min. The product was diluted 10-fold and used in nested PCR, which was performed using AP2 (provided with the kit) and gwUGT1-in as nested primers under the following conditions: 5 cycles at 94°C for 25 s and 70°C for 3 min; 30 cycles at 94°C for 25 s and 67°C for 3 min, and followed by 67°C for 7 min. For UGT94F2, gwUGT2-out and gwUGT2-in were used as primers for primary and nested PCR respectively, keeping rest of the conditions same as that for UGT86C4 promoter. The PCR products were purified and cloned into the *pTZ57R/T* cloning vector, and subsequently sequenced. Putative *cis*-acting elements upstream to start codon of the both UGTs were identified by searching PlantCare (http://bioinformatics.psb.ugent.be/webtools/plantcare/html/) and PLACE (http://www.dna.affrc. go.jp/PLACE/) databases.

### Quantitative Real-time PCR Analysis (qRT-PCR)

Total RNA from leaf, rhizome and inflorescence tissues were extracted as described above. DNA contamination was removed from the RNA samples using DNase I (50 U/µL, Fermentas, Burlington, Canada). RNA concentration was measured at 260 nm by a spectrophotometer (NanoDrop Technologies, Wilmington, DE, USA). The quality and purity of the preparations were determined at OD_260_:OD_280_ nm absorption ratio (1.8–2.0) and the integrity of the preparations was ascertained by electrophoresis using 1.2% agarose gel containing formaldehyde. About 2 µg of total RNA was used to synthesize first strand cDNA primed with OligodT in a 20 µL reaction mix using Revert-aid Premium M-MuLV reverse transcriptase (Fermentas, Burlington, Canada) following manufacturer’s instructions. Real-time PCR reactions were performed in triplicates using SYBR *Premix* Ex *Taq* (Takara, Dalian, China) in 48-well optical plates using ABI StepOne Real-time qPCR system (Applied Biosystems, Foster City, CA, USA). The PCR reaction (20 µL) included 0.2 µL cDNA template, 200 nM each of the primers, and 10 µL SYBR *Premix Ex Taq*. The cycling parameters were 95°C for 20 s, followed by 40 cycles of 95°C for 15 s and 60°C for 1 min. The specificity of each primer pair was validated by a dissociation curve (a single peak was observed for each primer pair). The primers used for real-time PCR analysis were designed with the help of Primer Express Version 3.0. (Applied Biosystems, Foster City, CA, USA). A constitutive active gene, β-actin was used as endogenous control (RtActinF and RtActinR).

To study the modulation of expression of UGTs by MeJA, SA and 2, 4-D, leaves were excised at different time intervals and analysed for UGT86C4 and UGT94F2 gene expression. The experiments were repeated thrice and the data were analysed statistically (*ANOVA*). The real-time PCR amplification data were exported into Microsoft Excel and gene expression levels were calculated based on the comparative C_t_ method [32].

### Extraction and Quantification of Picrosides Using HPLC

Plant samples of *P. kurrooa* were completely dried under gentle air stream (temperature 25±2°C and relative humidity 65±5%). The leaves, inflorescence as well as the rhizomes of the dried samples were ground separately into fine powder with a mortar and pestle. The powdered samples (10 g of each sample) were serially extracted with ethanol:water (1∶1) for 3 h at 25±2°C. The aqueous ethanolic extracts were filtered and dried by evaporation at reduced pressure using rotary evaporator at 40°C. The crude extracts of all the samples were dissolved in the methanol (20 mg/mL) and were filter sterilized with 0.22 µm membrane filters (Millipore, Bedford, USA). Stock solutions of the pure reference compounds (1 mg/mL) were prepared in HPLC grade water and stored in a refrigerator at 4°C. From the stock solutions, working solutions for each reference compound were prepared by dilution with HPLC grade water. These working solutions of both the reference compounds (5 µL of each) were mixed together in equal volumes for further analysis. A 10 µL aliquot of this solution was taken for the calibration curve. The HPLC (Shimadzu CLASS-VP V 6.14 SPI model) equipped with RP-18e column (E-Merck, 5 µm, 4.6×250 nm), a photo-diode array detector (SPD-M10A VP model) and a pump (LC-10AT VP model) was used for analysis of picrosides. The analysis was carried out using a mobile phase of methanol:water (2∶3) which was delivered at a flow rate of 0.7 mL/min. The detection wavelength was set at 270 nm. Injection volume of the sample was 10 µL and the column temperature was set at 30°C.

### Heterologous Expression of UGT94F2 and UGT86C4 in *E. coli*

The entire open reading frames of UGT86C4 and UGT94F2 were amplified using first-strand cDNA as the template with forward primers BAMUGT1F/BAMUGT2F harbouring a *Bam*HI restriction site and reverse primers NOTUGT1R/NOTUGT2R that included a *Not*I site. PCR was performed using Phusion high fidelity amplification system (New England Biolabs, Ipswich, MA, USA). The resulting PCR products were digested with *Bam*HI and *Not*I, gel purified, and ligated into the *Bam*HI and *Not*I digested *pGEX4T-2* vector (GE Lifesciences, Pittsburgh, PA, USA). The construct was sequenced to confirm that the gene was in the correct reading frame. The constructs, pGEX-UGT94F2 and pGEX-UGT86C4 were transformed into the *E. coli* strain *BL21* (DE3) according to the supplier’s recommendations (Novagen, Madison, WI). After transformation, colonies were selected on LB medium containing 100 µg/mL ampicillin. Individual positive colonies were grown overnight in 5 mL LB and 1% of overnight cultures were used to inoculate fresh LB and cells were grown at 37°C until the O.D of 0.5–0.6 was reached. 1 mM IPTG (Isopropyl β-D-1-thiogalactopyranoside) was added to the exponentially growing cells to induce the gene expression and 1 mL of induced cultures were harvested after every 2 h interval for 8 h. The harvested 1 mL culture samples were centrifuged at 13000 g for 5 min at 4°C. The cells were re-suspended in 3 mL of extraction buffer (50 mmol l^−1^Tris-HCl, pH-8.0, 10 mM MgCl_2_, 20% glycerol, 5 mM 2-mercaptoethanol) and disrupted by sonication on ice for 15–20 s burst time period. The lysate was centrifuged at 13000 g for 20 min at 4°C and the supernatant was loaded on 10% SDS-PAGE gel after heating the sample with 2 X SDS loading dye at 99°C for 10 min. Protein concentration was determined by Bradford assay using serum albumin as a standard.

## Results and Discussion

### Cloning and Sequence Analysis of UGT86C4 and UGT94F2

To identify glucosyltransferases involved in the iridoid O-glucosylation from *P. kurrooa*, a homology based strategy was employed taking advantage of the highly conserved amino acid motif among plant secondary product glycosyltransferases (PSPG box) located in the C-terminal region. Two forward degenerate primers were designed based on amino acid sequences F(L/V)(T/S)HCGWN and CGWNS(T/V)LE respectively in the conserved PSPG box of plant glucosyltransferases. Since there is only one conserved region in UGTs, we employed 3′ RACE strategy using a modified adapter-oligo-dT primer for the first strand cDNA synthesis. Partial cDNA fragments were amplified using the degenerate primers along with 3′ RACE adapter primer, and the fragments were cloned into *pTZ57R/T* cloning vector and analysed. Homology search using BLASTX algorithm revealed two partial cDNA fragments with their deduced amino acid sequences similar to the C-terminal sequences of various PSPGs in the database. Primers were designed using these two partial cDNA sequences and the full length cDNAs of both the glucosyltransferases were obtained using 5′ RACE PCR. The sequence information from 5′ and 3′ RACE allowed the design of gene specific primers to obtain the full-length transcripts.

The nucleotide sequence of full length cDNAs of both genes were submitted to NCBI GenBank under accession numbers JQ996408 and JQ996409. The amino acid sequences of both the cDNAs were named UGT86C4 (JQ996408) and UGT94F2 (JQ996409) as per the UGT nomenclature (http://www.flinders.edu.au/medicine/sites/clinical-pharmacology/ugt-homepage.cfm).

The full length cDNA of UGT86C4 (1641 bp) contains an open reading frame (ORF) of 1422 bp corresponding to a protein of 473 amino acids (Figure S1-A) with a calculated molecular weight of 53 kDa and an isoelectric point (pI) of 4.72. The full length cDNA of UGT94F2 (1612 bp) contains an ORF of 1455 bp encoding a protein of 484 amino acids (Figure S1-B) with a calculated molecular weight of 55 kDa and pI of 5.26. The UDP-glucosyltransferases UGT86C4 and UGT94F2 were identified from *P. kurrooa* based on the conservation of amino acid domains of plant UDP-glycosyltransferases associated with secondary metabolism [5], [33]. In the C-terminal domain, both UGT86C4 and UGT94F2 contain a motif that shows similarity with the 44-amino acid-long PSPG sequence found in most known UDP-glycosyltransferases. The motif comprises amino acid residues playing a key role in binding the nucleotide-diphosphate sugar [34]. Particularly, Trp (W), Glu/Asp (D/E), and Gln (Q) residues found in the PSPG sequence of both the UGTs (Figure S2; at positions 22, 43, and 44 of the motif, respectively) have been shown to be conserved in several plant UDP-glucosyltransferases, preferring UDP-Glc as the sugar donor substrate [35].

Analysis of UGT86C4 and UGT94F2 sequence for N-terminal targeting signal or C-terminal membrane anchor signal using the Phobius Web-based program predicted trans membrane N-terminal domain for UGT94F2 (Figure S3), whereas very weak signal peptide or trans-membrane domain was found for UGT86C4 (Figure S3). Furthermore, UGT86C4 and UGT94F2 were also analysed for the presence of glycosylated residues using the NetNGlyc 1.0 server. This program predicted the presence of two putative glycosylation sites in two Asn residues: Asn-247 (NFSK) and Asn-443 (NATQ) for UGT86C4 (Figure S4) and three putative glycosylation sites in three Asn residues: Asn-35 (NFTV), Asn-204 (NLSS) and Asn-213 (NSSK) for UGT94F2 (Figure S4).

### Phylogenetic Analysis of UGT94F2 and UGT86C4

**Figure 2:**
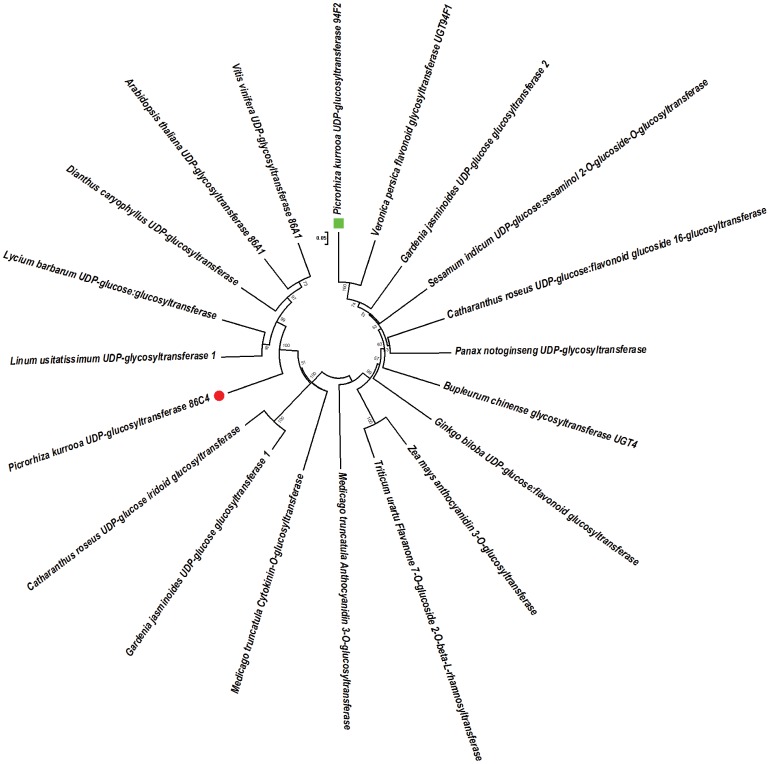
Phylogenetic tree of deduced amino acid sequences of UGT86C4 and UGT94F2. Phylogeny of UGTs was inferred using neighbour-joining method of the MEGA 5.1 software. A total of 20 protein sequences used for analysis were from following plant species: *Picrorhiza kurrooa* UGT94F2 (JQ996409), *Picrorhiza kurrooa* UGT86C4 (JQ996408), *Arabidopsis thaliana* UDP-glycosyltransferase 86A1 (AAM91353), *Linum usitatissimum* UDP-glycosyltransferase 1 (AFJ53011), *Dianthus caryophyllus* UDP-glucosyltransferase (BAF75888), *Lycium barbarum* UDP-glucose:glucosyltransferase (BAG80548), *Gardenia jasminoides* UDP-glucose glucosyltransferase (BAK55737) *Catharanthus roseus* UDP-glucose iridoid glucosyltransferase (BAK55749), *Medicago truncatula* cytokinin-O-glucosyltransferase (XP_003615832), *Veronica persica* flavonoid glycosyltransferase UGT94F1 (BAI44133), *Gardenia jasminoides* UDP-glucose glucosyltransferase (BAM28984), *Catharanthus roseus* UDP-glucose:flavonoid glucoside 1,6-glucosyltransferase (BAH80312), *Panax notoginseng* UDP-glycosyltransferase (AED99884), *Sesamum indicum* UDP-glucose:sesaminol 2′-O-glucoside-O-glucosyltransferase (BAF99027), *Bupleurum chinense* glycosyltransferase UGT4 (AFK79036), *Zea mays* anthocyanidin 3-O-glucosyltransferase (ACG26485), *Ginkgo biloba* UDP-glucose:flavonoid glucosyltransferase (AEQ33588), *Triticum urartu* flavanone 7-O-glucoside 2′′-O-beta-L-rhamnosyltransferas (EMS55799), *Medicago truncatula* anthocyanidin 3-O-glucosyltransferase (AES75297), *Vitis vinifera* UDP-glycosyltransferase 86A1(XP_002276858).

Pairwise alignment of deduced primary structures of UGT94F2 and UGT86C4 showed that these are highly diverse, with only 22% identical at amino acid level and 47.8% identical at the nucleotide level. To ascertain the degree of evolutionary relatedness, Neighbour-joining phylogenetic tree was constructed with MEGA 5.10 software from the ClustalW alignment of UGT94F2 and UGT86C4 with number of UGT orthologs from different plants retrieved from the NCBI GenBank database. As shown in phylogenetic tree (Figure 2), UGT86C4 showed the highest amino acid sequence identity to other plant UDP-glycosyltransferases including *Arabidopsis thaliana* AAM91353 (47%), *Linum usitatissimum* AFJ53011 (47%), *Dianthus caryophyllus* BAF75888 (46%), *Lycium barbarum* BAG80548 (44%), *Gardenia jasminoides* BAK55737 (33%), *Catharunthus roseus* BAK55749 (32%) and *Medicago truncatula* XP_003615832 (32%). UGT94F2 showed the highest amino acid sequence identity to other orthologs from *Veronica persica* BAI44133 (53%), *G. jasminoides* BAM28984 (47%), *C. roseus* BAH80312.1 (42%), *Prunus persica* AEJ88222.1 (41%) and *Panax notoginseng* AED99884.1 (40%). In case of glycosyltransferases, the phylogenetic relationships are not always an indicative of the possible role of these enzymes since the relationship between the degree of amino acid sequence identity and substrate specificity of the plant UGTs is highly complex. It has been observed that appreciably diverse UGTs often share the same substrates. It is amply illustrated by the two *Dorotheanthus bellidiformis* UGTs, UGT73A5 and UGT71F2. There is 20% amino acid sequence identity between these two UGTs [36] but same set of acceptor molecules are glucosylated by these UGTs [37]. On the contrary, two *Allium cepa* UGTs designated AcUGT73G1 and AcUGT73J1 belong to the same UGT family but with different range of specificities. AcUGT73G1 enzyme exhibits very broad acceptor specificity whereas AcUGT71J1 has a very narrow specificity [38]. Furthermore, *M. truncatula* UGT71G1 and *Arabidopsis* UGT71C1 have glucosylation activity towards triterpene and coumarin *in vitro*, respectively [39] while as *Arabidopsis* UGT71B proteins are able to glucosylate a range of different compounds such as abscisic acid and hydroxybenzoic acid [40].

Because sequence homology is far from being a definitive argument to describe the precise enzymatic activity of glycosyltransferase enzymes [41], the functional characterization of these enzymes is still necessary. Furthermore, correlations between transcripts and metabolites could facilitate an efficient narrowing down of candidate products of glycosyltransferase activities [42], [43].

### Protein 3-D Structure Prediction and Structural Insight into UGT86C4 and UGT94F2

**Figure 3:**
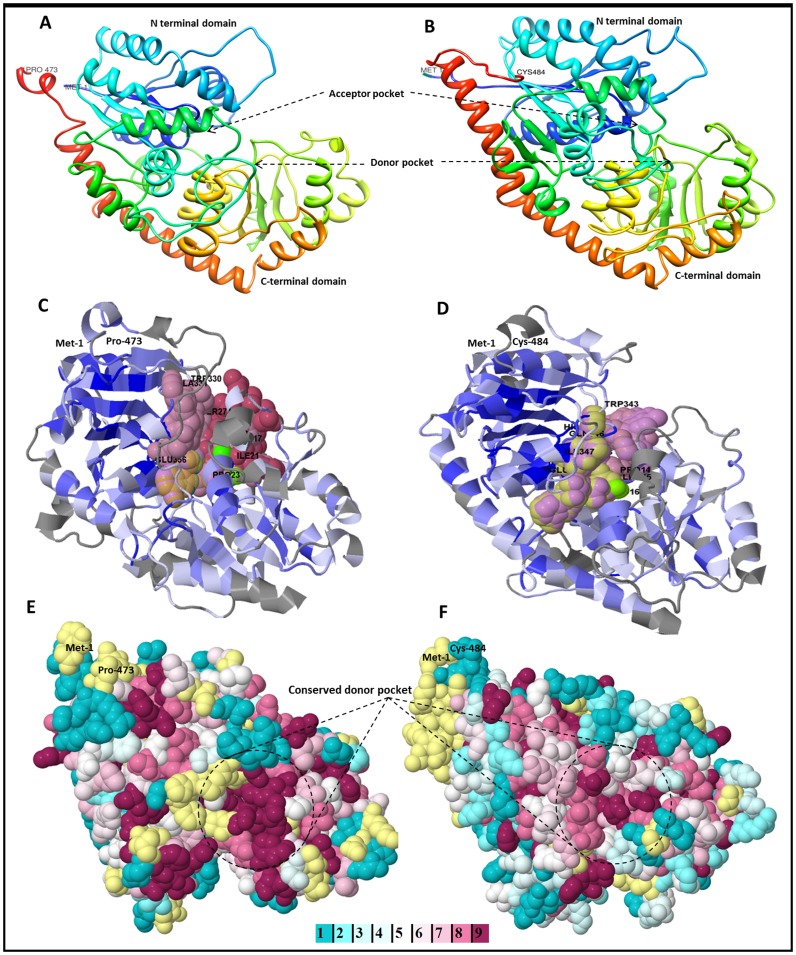
Three dimensional models and conserved residue prediction for UGT86C4 and UGT94F2. A and B: Ribbon display of the 3-D structures of UGT86C4 and UGT94F2 as predicted by PHYRE2 web server, using crystal structure *Arabidopsis thaliana* UGT72B1 (Protein Data Bank (PDB) Id. 2VCH-A) as template for modeling of both the proteins. N and C-terminal domains are shown. Donor and acceptor sites are also shown. C and D: Predicted ligand binding sites as predicted by 3DLigandSite web server. E and F: Evolutionary conserved residue analysis of UGT86C4 and UGT94F2 were performed using Consurf, an empirical Bayesian inference based web server. Residue conservation from variable to conserved is shown in blue (1) to violet (9). The residues involved in binding of the donor moieties are shown in the centre of the structures.

Functional characterization of UGTs requires time demanding biochemical studies as well as the study of site-specific mutants. Molecular modeling is becoming an important tool to guide the biochemical and functional characterization of the large number of UGTs, as well as facilitating UGT enzyme engineering and metabolic engineering of crop plants. The number of UGTs for which the X-ray crystal structure has been solved is limited in comparison to the rate of appearance of new UGT sequences. This reflects the difficulties in obtaining well diffracting crystals and in solving the structures [44]. To circumvent this bottleneck in the study of UGTs, homology modeling has proved to be a strong tool for the identification of potentially important residues [45], [46]. The high conservation of secondary and tertiary structures among the UGTs prompts homology modeling as an attractive tool for studying their substrate specificity as well as other structure derived properties. The recently solved crystal structures of five plant UGTs including *M. truncatula* UGT71G1 [35], UGT85H2 [47] and UGT78G1 [48]; *Vitis vinifera* VvGT1 [6]; and *A. thaliana* UGT72B1 [49] essentially add confidence to the structures derived from homology modeling. To understand the basis for the substrate specificity of UGT86C4 and UGT94F2, protein homology models were constructed using PHYRE2 web server with the crystal structure of *Arabidopsis* UGT72B1 (pdb id. 2VCH-A) as the template (Figure S5). Although this procedure does not necessarily describe the real three dimensional structure of the protein, the plant UGT structures are highly similar to each other though the enzymes share relatively low sequence identities. The proposed model for *Picrorhiza* UGT86C4 and UGT94F2 shares the structural features described for the Family-1 UGTs by adopting the so-called GT B-fold formed by the C-terminal and N-terminal domains separated by an inter-domain linker and consists of two N- and C-terminal domains with similar Rossmann-like folds [34]. The sugar donor is deeply buried in a narrow groove in the C-terminal domain and interacts with the highly conserved PSPG motif and the acceptor mainly binds to the N-terminal domains (Figure 3A and 3B).

The stereo-chemical qualities of the predicted models of UGT86C4 and UGT94F2 proteins were validated by PROCHECK server. Ramachandran plot analysis of UGT86C4 showed 86.5% residues in the most favourable region, 11.4% residues in the additional allowed region, 1.0% in the generously allowed region and 1.2% in the disallowed region (Figure S6-A). Whereas for UGT94F2 model, it showed 88.2% residues in the most favourable region, 9.6% residues in the additional allowed region, 1.2% residues in the generously allowed region and 1.0% in the disallowed region (Figure S6-B).

The results of the PROCHECK analysis indicate that a relatively low percentage of residues have phi/psi angles in the disallowed regions suggesting the acceptability of Ramachandran plots for UGT proteins. The percentage of residues in the allowed region were found to be 98.2 and 99% for UGT86C4 and UGT94F2 respectively, while residues in disallowed regions were found to be 1.2% and 1% for UGT86C4 and UGT94F2 respectively.

The energy refined models of both the *Picrorhiza* UGTs were submitted to the public domain PMDB database (http://www.caspur.it/PMDB/) and assigned PMDB IDs, i.e., PM0078442 and PM0078441 for UGT86C4 and UGT94F2 respectively. Successfully modeled structures of UGT86C4 and UGT94F2 (PM0078442 and PM0078441) were subjected to PDBsum server for structural motif analysis. The presence of α-helix, β-sheets, turns, and coil were frequently observed in both the UGT proteins. UGT86C4 consists of 3 β-sheets, 6 β-α-β units, 1 β-hairpin, 12 strands, 21 helices, 25 helix-helix interacs, 46 β-turns and 12 γ-turns. UGT94F2 consists of 2 β-sheets, 8 β-α-β units, 1 β-bulge, 12 strands, 20 helices, 24 helix-helix interacs, 46 β-turns and 15 γ-turns (Figure S7-A and B).

The amino acid residues involved in ligand binding were also predicted using the 3DLigandSite tool as depicted (Figure 3C and 3D). Analysis of the evolutionary conservation of UGT86C4 and UGT94F2 amino acids was performed using ConSurf program. Several residues with high scores were found to be functional and structural residues in the proteins. Particularly the C-terminal PSPG box was found to be highly conserved across all major plant UGTs (Figure 3E and 3F).

### Molecular Docking and Acceptor Substrate Recognition for UGT86C4 and UGT94F2

Acceptor substrates are much more complex and diverse, and require different UGTs with different topologies of binding pockets. The acceptor binding pocket is adjacent to the donor binding site, mainly consisting of residues in the N-terminal domain and some residues in the C-terminal domain, formed by several helices and loops (Figure 3A and 3B). These regions are highly varied in these UGTs, including the sequence length and amino acid composition.

**Figure 4:**
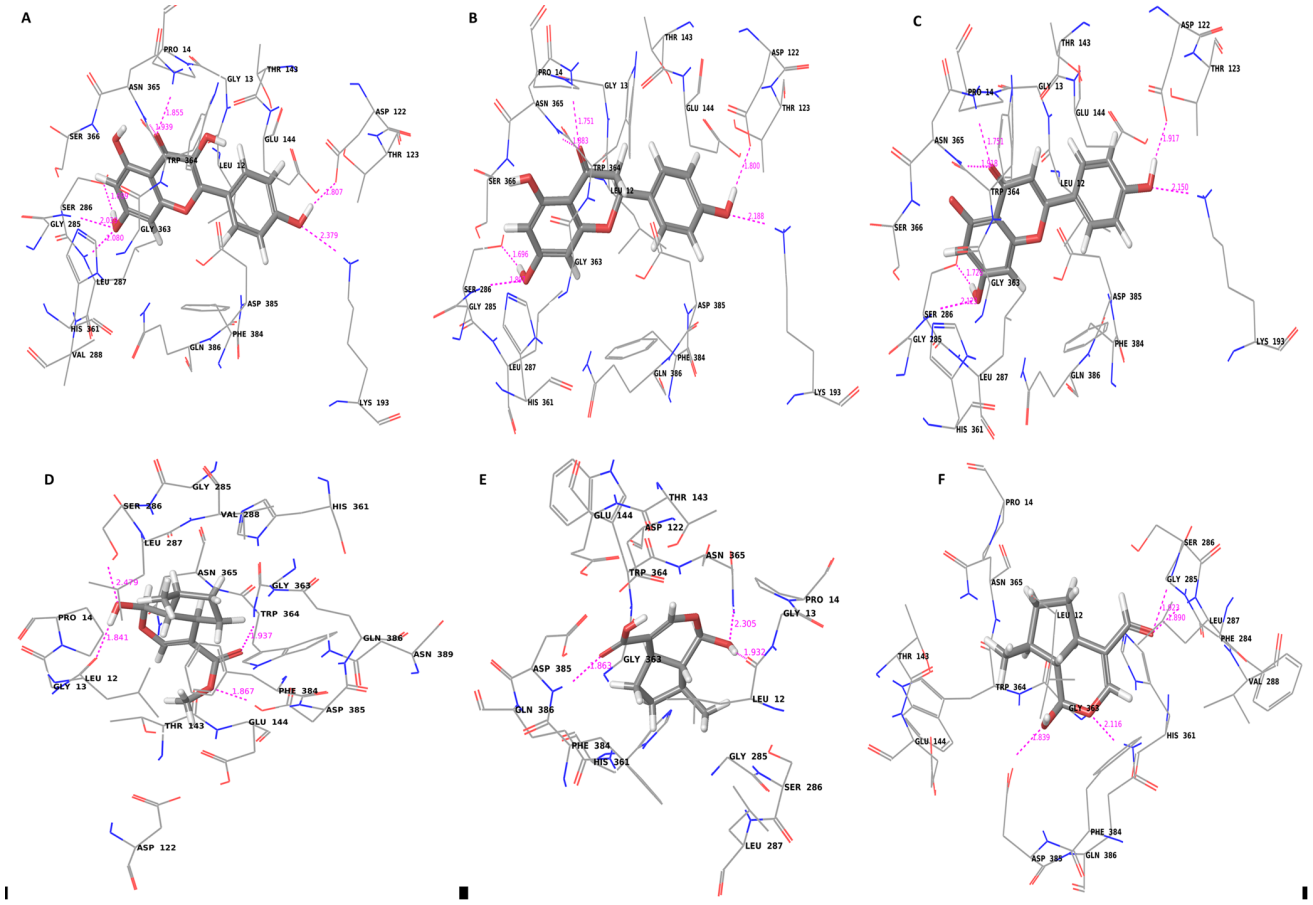
Molecular docking of UGT86C4. Diagram showing (A) kaempferol (B) naringenin (C) apigenin (D) 7-deoxyloganetin (E) 7-deoxyloganetic acid and (F) iridotrial docked into the proposed binding pockets of UGT86C4.

**Figure 5:**
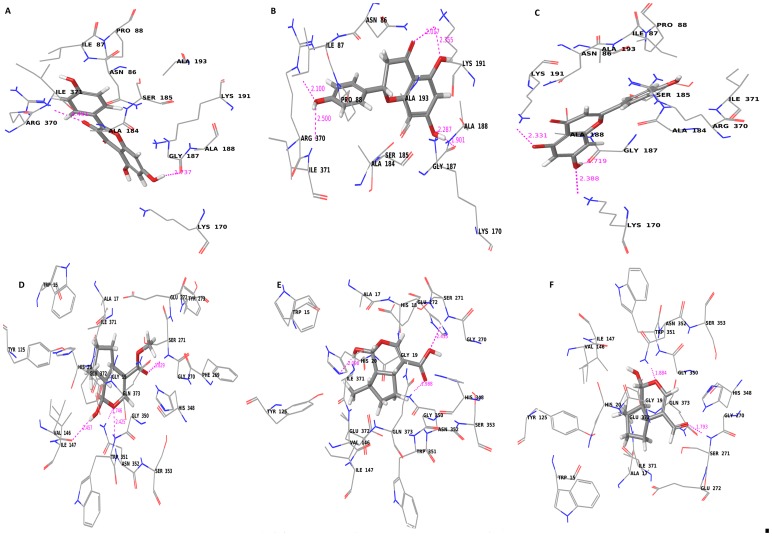
Molecular docking of UGT94F2. Diagram showing (A) kaempferol (B) naringenin (C) apigenin (D) 7-deoxyloganetin (E) 7-deoxyloganetic acid and (F) iridotrial docked into the proposed binding pockets of UGT94F2.

**Figure 6:**
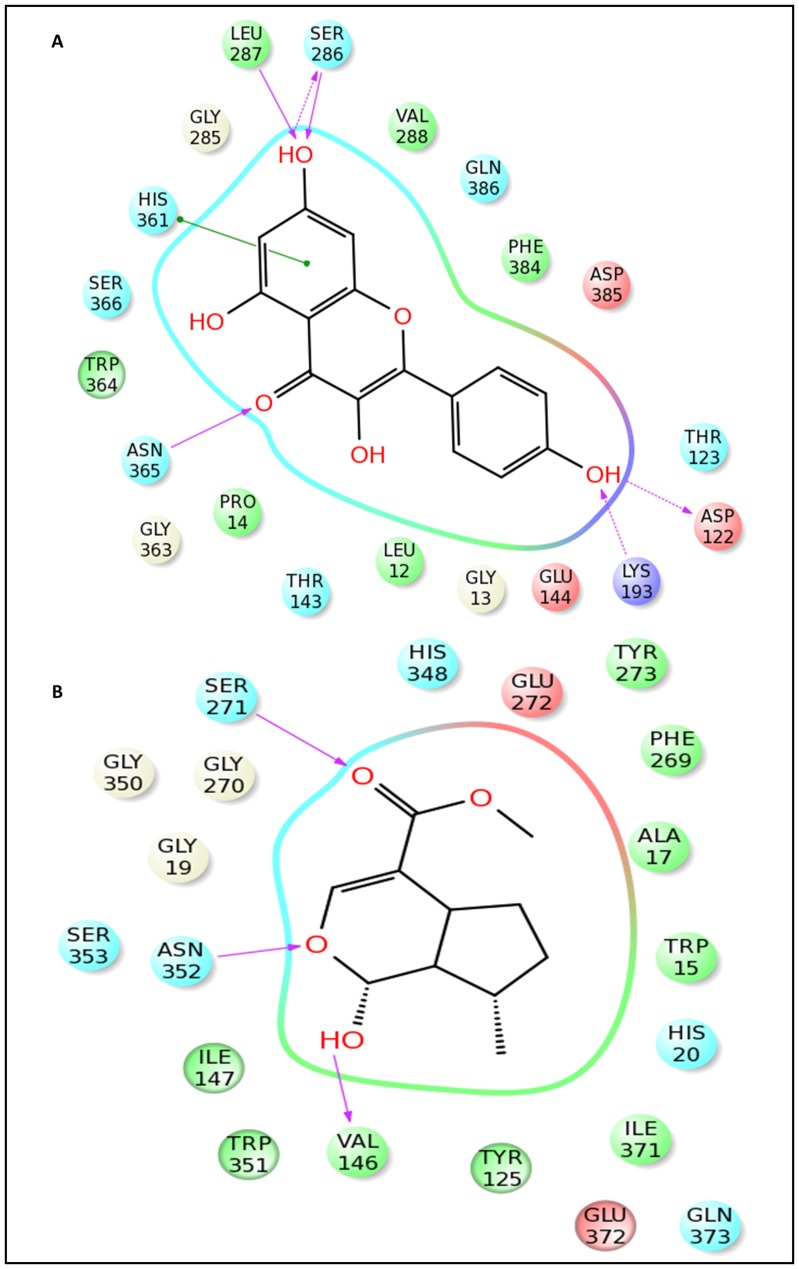
2-D representation of interactions of UGT86C4 and UGT94F2. 2-D representation of the interaction figure (in pink) of kaempferol with UGT86C4 (A), and 2-D interaction figure of 7-deoxyloganetin with UGT94F2 (B).

Docking experiments were initially performed using iridotrial, 7-deoxyloganetic acid, 7-deoxyloganetin, apigenin, kaempferol and naringenin (Figure 4 and 5; Figure S8). Based on the docking and Prime MMGBSA results of UGT86C4, the docking studies of the ligand dataset i.e., iridotrial, 7-deoxyloganetic acid, 7-deoxyloganetin, apigenin and naringenin showed similar binding affinity except for kaempferol where the binding affinity was comparatively higher (Table 1). Kaempferol is quite suitably placed within the proposed binding pocket of UGT86C4 as shown in Figure 4A. It is proposed to involve in five H-bond formation with Asp122 (1.81 Å), Lys193 (2.38 Å), Ser286 (1.7 Å and 2.04 Å), Leu287 (2.08 Å) and Asn365 (1.85 Å and 1.94 Å) (Figure 6A). UGT94F2 was found to have strong binding affinity for iridoid class of compounds including iridotrial, 7-deoxyloganetic acid, 7-deoxyloganetin in comparison to apigenin, kaempferol and naringenin (Figure 5). Out of the three iridoid moieties, UGT94F2 showed maximum binding affinity towards 7-deoxyloganetin. The compounds apigenin, kaempferol and naringenin showed very poor binding affinity towards the protein’s acceptor site both in terms of dock score and binding energy parameters (Table 1). The interaction of 7-deoxyloganetin, having the best binding affinity as per the docking studies, within the acceptor site of UGT94F2 is shown in (Figure 5D). It is proposed to be involved in three H-bonds with Val146, Ser271 and Asn352. The oxygen of the pyran ring is involved in two H-bonds with Asn352 at 1.746 Å and 2.425 Å and the hydroxyl group attached to the pyran ring is involved in H-bond with Val146 at 2.457 Å. Besides this, the oxygen of the carboxyl group is involved in H-bond with Ser271 at 1.829 Å. These four H-bonds appears to provide stability to the molecule within the binding pocket. It was also revealed that Ser271 and Asn352 plays pivotal role in substrate identification (Figure 6B).

**Table 1:** Binding affinity of ligand data set on modeled structures of UGT86C4 and UGT94F2.

S. No.	Ligand	MMGBSAdG Bind	
		UGT94F2	UGT86C4
1.	Kaempferol	−42.501	−72.520
2.	Naringenin	−37.472	−69.824
3.	Apigenin	−43.278	−60.900
4.	7-deoxyloganetin	−95.978	−59.780
5.	7-deoxyloganetic acid	−65.150	−53.886
6.	Iridotrial	−64.297	−50.505

Our modeling studies are in conformity with the earlier findings wherein an iridoid-specific glucosyltransferase from *G. jasminoides* was found to preferably glucosylate 7-deoxyloganetin in comparison to iridotrial, 7-deoxyloganetic acid or loganetin [29]. One can possibly infer that in *Picrorhiza*, UGT94F2 may also preferentially glucosylate the 1-O-hydroxyl group of 7-deoxyloganetin.

### Donor Recognition and Sugar Specificity of UGT86C4 and UGT94F2

UGTs recognizing several different sugar donors have been identified. These include UDP-Glc, UDP-Gal, UDP-Xyl, UDP-Rha, and UDP-GlcUA. In plant UGTs, the most common sugar donor is UDP-Glc. Several conserved residues, most of which are found in the PSPG motif of plant UGTs, interact with the sugar donor [6], [35], [46], [47]. The sugar donor preference of a specific UGT is often very narrow, showing little or no activity with alternative sugars. In the crystal structures of UGTs, the UDP-glucose sugar donor is located in a long, narrow channel mainly within the C-terminal domain of the enzymes. It mainly interacts with residues in the plant UGT signature PSPG motif which has a highly conserved consensus sequence with 44 amino acids. The glucose moiety of sugar donor mainly interacts with the last two residues of the PSPG motif (Glu381 and Gln382 in UGT71G1) and another highly conserved tryptophan (Trp360 in UGT71G1) forming hydrogen bonds with its 2-, 3- and 4-OH [47].

The deduced amino acid sequence of UGT86C4 and UGT94F2 had the important conserved amino acid residues (His-12 and Asp-115, UGT94F2; His-20 and Asp-124) (Figure S2). Noguchi et al. [50] demonstrated that substitution of the Arg with Trp residue in the PSPG box was sufficient to convert donor specificity from UDP-glucuronic acid to UDP-glucose flavonoid 7-O- glucuronosyltransferase (F7GAT) in Lamiales. The corresponding residue of UGT86C4 and UGT94F2 is proline (Pro-15 in UGT86C4 and Pro-23 inn UGT94B2), as is the case with *V. vinifera* VvGT1 (Pro-23) *A. thaliana* AtUGT72B1 (Pro-22), *G. jasmonides* UGT85A24 (Pro-25) and *C. roseus* UGT85A23 (Pro-28), all known to use UDP-glucose as sugar donor and act as UDP-glucosyltransferases (Figure S2). Therefore, the *Picrorhiza* UGTs, UGT86C4 and UGT94F2 belong specifically to UDP-glucosyltransferase group.

### Promoter Isolation and Identification of *cis-*regulatory Elements

Variation in the picroside accumulation pattern in different organs could be mainly because of the differences in the *cis*-regulatory region of the important pathway genes. In *Arabidopsis*, there is clear evidence that R2R3-MYB transcription factors are able to activate glycosyltransferase expression [51], controlling flavonol accumulation in an organ-specific and development-dependent manner. Hence, to elucidate whether the differential expression patterns of the two *Picrorhiza* UGT genes correlates with transcriptional regulation *via* their promoters, upstream region of both the UGT genes were isolated and scanned for putative *cis*-regulatory elements using the PlantCARE and PLACE databases.

**Figure 7:**
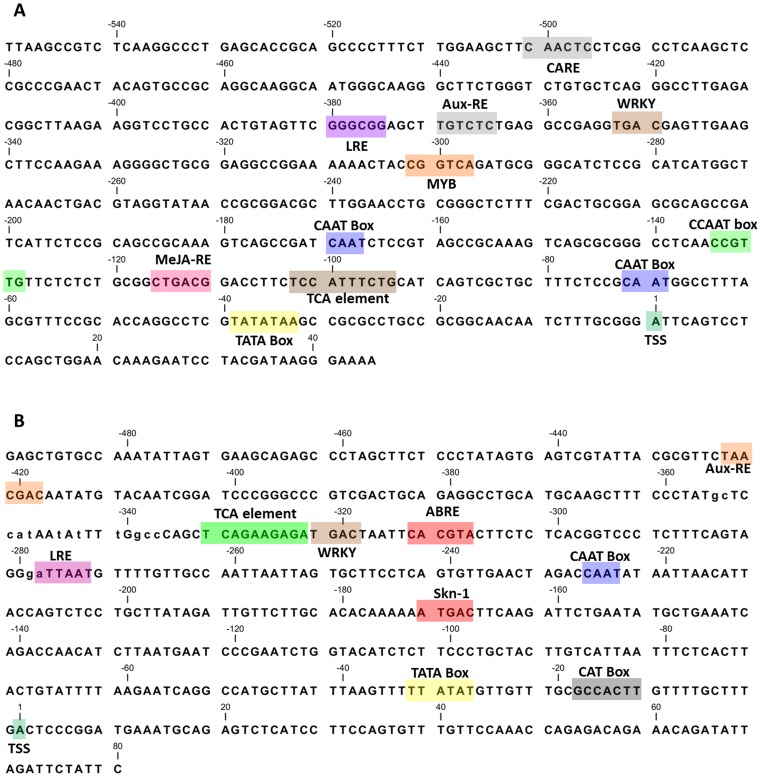
Nucleotide sequences of *Picrorhiza* UGT gene promoters. Nucleotide sequences of the UGT86C4 (A) and UGT94F2 (B) gene promoters. Numbering starts from the predicted transcription start site (dark green shaded). The putative core promoter consensus sequences and the motifs with significant similarity to the previously identified *cis*-acting elements are shaded and the names are given.

Using genome walking strategy, we isolated a 595 bp and 571 bp 5′ upstream regions of UGT86C4 and UGT94F2 genes. The predicted transcription start site (TSS, +1) correlated well with the 5′ RACE analysis and is located at 45 bp upstream in case of UGT86C4 and 80 bp upstream in case of UGT94F2, with respect to the ATG start codon (Figure 7A and 7B). Both the promoter regions possessed a typically high A+T content (65.1% for UGT86C4 and 62.3% for UGT94F2) commonly found in other plant promoters. Computational analysis using PlantCare and PLACE databases revealed several *cis-*elements including eight potential TATA box sequences within the promoter region of both the genes (UGT86C4 at positions −32, −251, – 35, +33 and −250; UGT94F2 at positions −25, +30, −27, +219, +444, −193, −26, +431, −60, +28 and +218). Another consensus eukaryotic *cis*-element CAAT box, was also observed at positions −9, −166, −68, −448, −166, −68 and −448 in case of UGT86C4, while in case of UGT94F2, CAAT box was observed at −188, −405, −257, +477, −223 and +258. Scanning of promoter boxes also resulted in the identification of several *cis*-elements including numerous light-responsive elements, some sugar-responsive elements, hormone-responsive elements, stress-responsive elements, and a meristem-specific activation element (Figure 7; Table 2). Bioinformatic analysis also revealed the presence of several motifs essential for tissue-specific expression and other putative *cis*-acting elements involved in different biological processes such as MYB-binding site and W-box recognizing WRKY-type transcription factors.

**Table 2:** Putative *cis*-acting regulatory elements identified in the promoter of UGT86C4 and UGT94F2, using PLACE (http://www.dna.affrc.go.jp/PLACE) and PlantCare databases (http://bioinformatics.psb.ugent.be/webtools/plantcare/html/).

	Position				
Site Name	UGT86C4	UGT94F2		Sequence	Function
CAAT-box	−9, −166, −68, −448, −166, −68, −448		−188, −405, −257, +477, −223, +258	CAAT	common *cis*-acting element in promoter and enhancer regions
CAT-box	−387		−12	GCCACT	*cis*-acting regulatory element related to meristem expression
CCAAT-box	−128		−142	CAACGG	MYBHv1 binding site
CGTCA-motif	−110, −348, −259		− 54, +81	CGTCA	*cis-*acting regulatory element involved in the MeJA-responsiveness
GAG-motif	−281		−101, +320	GGAGATG	part of a light responsive element
Gap-box	−96		–	AAATGGAGA	part of a light responsive element
MBS	−262, −296		–	CAACTG	MYB binding site involved in drought-inducibility
Sp1	374, −475		–	GGGCGG	light responsive element
TATA-box	−32, −251, −35+33, −250		−25, +30, −27, +219, +444, −193, +431, −60, +28,+218	TATA	core promoter element around −30 of transcription start
TCA-element	−93		+322	CAGAAAAGGA	*cis*-acting element involved in salicylic acid responsiveness
TGACG-motif	−110, +348, −259		–	TGACG	*cis*-acting regulatory element involved in the MeJA-responsiveness
ABRE	–		−307	TACGTG	*cis*-acting element involved in the abscisic acid responsiveness
Box 4	–		−80, +272, −254	ATTAAT	part of a conserved DNA module involved in light responsiveness
G-Box	–		−307, +306	CACGTA	*cis*-acting regulatory element involved in light responsiveness
Skn-1_motif	–		−84, +318, −167	GTCAT	*cis*-acting regulatory element required for endosperm expression
TCCC-motif	–		447	TCTCCCT	part of a light responsive element
TGA-element	−417		–	AACGAC	auxin-responsive element
circadian	–		−399	CAANNNNATC	*cis*-acting regulatory element involved in circadian control
MYBCORE	−129, −132		−379	YAACKG	MYB recognition site found in genes that are responsive to water stress flavonoid biosynthesis
CGCG box	−17, −26		−136	VCGCGB	core of TGAC-containing W-box recognized by AtSR1-6 (*Arabidopsis))thaliana* signal-responsive genes)
W box	−125, −69		−185,+294	TGACT	binding site for WRKYs, possibly involved in elicitor-responsiveness transcription of defense genes
LTR	–		−437, +412	CCGAAA	*cis*-acting element involved in low-temperature responsiveness

### Tissue-specific Expression Analysis Using Quantitative Real-time PCR

**Figure 8:**
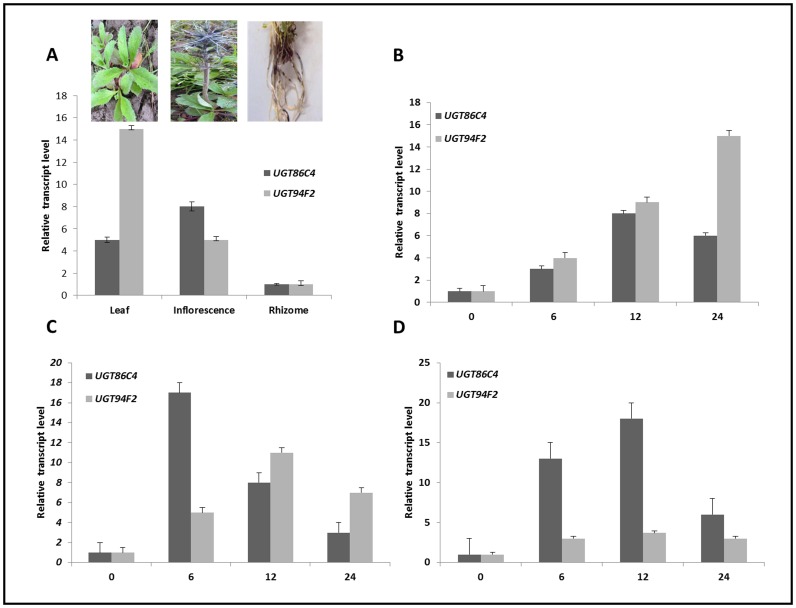
Tissue-specific real-time expression analysis and time-course effect of elicitor treatments on UGT86C4 and UGT94F2. Tissue-specific expression of UGT86C4 and UGT94F2 (A), time-course effect of methyl jasmonate (MeJA) (B), salicylic acid (SA) (C) and 2, 4-dichlorophenoxyacetic acid (2,4-D) (D) on the expression of UGT86C4 and UGT94F2. Data were compared and analysed with analysis of variance (*ANOVA*) test. Values are means, with standard errors indicated by bars, representing three independent biological samples, each with three technical replicates. Differences were scored as statistical significance at the *P<0.05 and **P<0.01 levels.

To investigate the expression profiles of UGT86C4 and UGT94F2 in different tissues of *P. kurrooa*, total RNA was isolated from rhizomes, leaves and inflorescence and subjected to qPCR analysis using gene specific primers (Table S1). UGT86C4 showed highest expression in inflorescence followed by leaves and least expression was found in roots. However, spatial expression pattern of UGT94F2 showed maximum transcript abundance in leaves, followed by inflorescence and least expression was found in rhizomes (Figure 8A). These results suggest that UGT86C4 and UGT94F2 are expressed differentialy in different plant organs hinting towards their spatial regulation.

### Effect of Elicitors on the Expression of UGT86C4 and UGT94F2

Several studies have shown that UGTs are induced by a variety of stresses, including SA [52], auxins [53], MeJA [54] and wounding [55]. To elucidate the role of various phytohormone treatments vis-à-vis the expression of UGT86C4 and UGT94F2 and to understand the regulatory role of the UGTs in *P. kurrooa* in relation picrosides accumulation, the effect of elicitors which included MeJA, SA and 2, 4-D was analysed. The results showed that the expression levels of both the UGTs were strongly increased by phytohormone treatments, though the expression pattern was different for different phytohormones (Figure 8).

MeJA is known to induce the biosynthesis of many secondary metabolites that play important role in the adaptation of plants to biotic stress [56]. In *C. roseus*, accumulation of monoterpene indole alkaloids (MIA) is induced in a 2, 4-D-free medium and can be further increased by the addition of MeJA [57]. Methyl jasmonate has been found to stimulate the biosynthesis of alkaloids by inducing the expression of several genes from the monoterpenoid branch of the MIA biosynthetic pathway [58]. MeJA also induces the expression of the *C. roseus* ORCA3, a transcription factor controlling many MIA biosynthetic genes [59]. In the present study, MeJA up-regulated the expression of both UGTs but at different time intervals after induction. UGT86C4 showed 8-fold increase in expression after 12 h of application of MeJA while UGT94F2 showed 15-fold increase in the expression after 24 h of MeJA application (Figure 8B).

With SA, our study shows differential transient increase in accumulation of UGT86C4 and UGT94F2. UGT94F2 showed maximum increase of 10-fold after 12 h of induction which corroborated well with the picroside accumulation. While as UGT86C4 showed 16-fold increase in the expression profile 6 h post induction (Figure 8C). The results are fully in consonance with the previous studies wherein genes that glycosylate flavonoids, phenylpropanoids and benzoic acid derivatives have been found to show an early response to SA treatment [60]. This further corroborates that UGT86C4 may be a flavonoid-specific glucosyltransferase as predicted by our modeling studies. Exogenous application of 2, 4-D resulted in increasing the expression of UGT86C4 but had rather less effect on the expression of UGT94F2 (Figure 8D). UGT86C4 showed maximum response of about 20-fold after 12 h of induction and there was only 3-fold increase in UGT94F2 expression. 2, 4-D has been found to have a down-regulatory effect on the iridoid pathway of MIA biosynthesis [57]. However in *P. kurrooa*, 2, 4-D increased the picroside levels, although the increase was less as compared to the effect of MeJA or SA.

### Tissue-specific Picroside Accumulation and Effect of Elicitors on Picroside Biosynthesis

**Figure 9:**
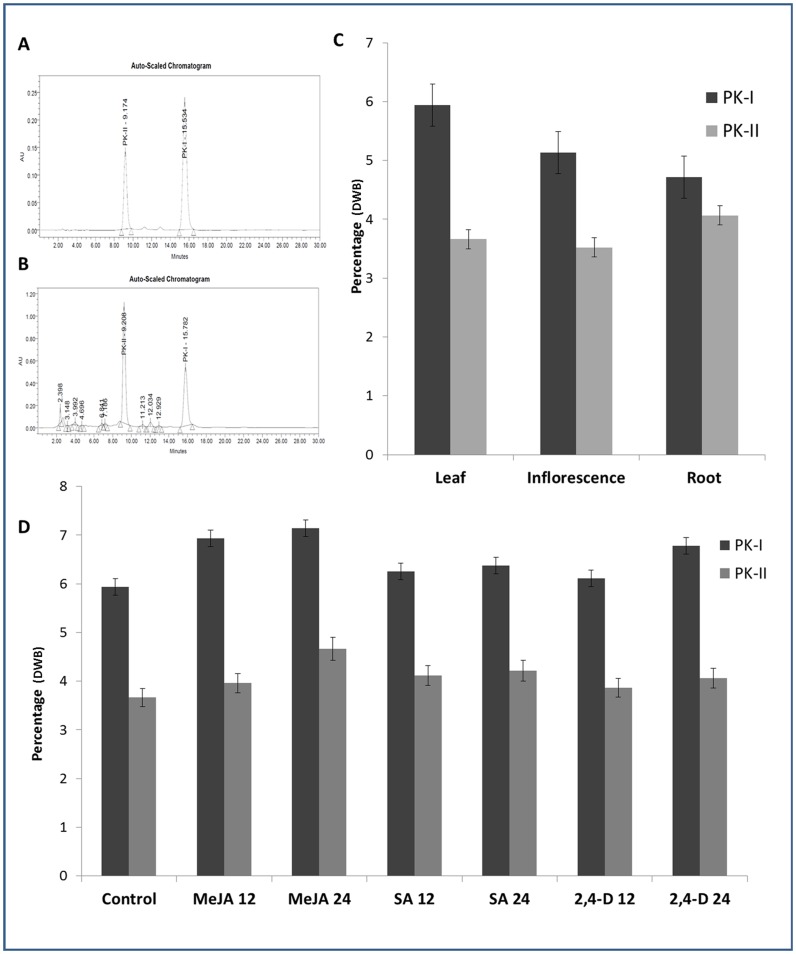
Tissue-specific and time-course effect of elicitor treatments on picroside accumulation. HPLC chromatogram of standard (picroside I and picroside II) at 270 nm (A). HPLC chromatogram of leaf sample showing peaks of picroside I and picroside II (B). Tissue-specific accumulation of picrosides (PK-I and PK-II) in leaves, inflorescence and rhizomes of *P. kurrooa* (C). Effect of methyl jasmonate (MeJA) (B), salicylic acid (SA) (C) and 2, 4-dichlorophenoxyacetic acid (2, 4-D) (D) treatments on picrosides accumulation at different time intervals (D). HPLC analysis demonstrated the change in two key picrosides, PK-I and PK-II at 12 and 24 h after treatments of mature leaves. All values obtained were means of triplicate with standard errors. Time course accumulation of PK-I and PK-II was statistically significant at p<0.001 level.

To corroborate the expression pattern of UGT86C4 and UGT94F2 with the level of picroside accumulation in different tissues of *P. kurrooa* including rhizomes, leaves and inflorescence were analysed for picroside accumulation using high performance liquid chromatography (Figure 9). The picroside contents were found to vary in different tissues with leaves having maximum accumulation of picroside-I (PK-I; 5.937±0.20% DWB) followed by inflorescence (5.139±0.12% DWB) and least accumulation was found in rhizomes (4.716±0.09% DWB). However, a different trend was seen in the levels of picroside-II, wherein roots showed highest levels (PK-II; 4.064±0.12% DWB) followed by leaves (3.6615±0.12% DWB) and least accumulation was found in case of inflorescence (3.521±0.12% DWB).

To comprehend the role of glucosyltransferases in the biosynthesis of picrosides and to establish a correlation between the change in picroside flux and the change in gene expression of glucosyltransferases (UGT86C4 and UGT94F2), the effect of MeJA, SA and 2, 4-D treatments on the biosynthesis of a key picrosides was analysed. These elicitors have been found to modulate the important secondary metabolic pathways [61]. In MeJA treated plants, there was an increase in the picrosides content (7.13±0.21% DWB) after 24 h compared to the untreated control (5.937±0.12% DWB). Similar results were also observed with 0.1 mM SA. With 2, 4-D, phytohormone known for inducing cell division and other growth related physiological phenomenon in plant cells, there was only a slight increase in picroside content, corroborating well with the expression of UGT94F2 that showed only a 3-fold increase in transcript levels (Figure 9).

Interestingly, the change in picroside flux upon elicitor treatment corroborated well with the change in expression profile of UGT94F2 (Figure 8). All these results tend to be in agreement with the UGT94F2 promoter analysis and indicate that UGT94F2 is signalling-responsive gene which may be involved in the glycosylation of iridoid moieties of picroside biosynthetic pathway secondary metabolites in *P. kurrooa.* These results are further endorsed by the molecular docking studies, wherein iridoid moieties especially 7-deoxyloganetin presented high binding affinity of towards UGT94F2.

### Heterologous Expression of Recombinant UGT86C4 and UGT94F2 in *E. coli*

The full length ORF of UGT86C4 and UGT94F2 were digested from the pJET-UGT86C4 and pJET-UGT94F2 gene constructs using *Bam*HI and *Not*I restriction enzymes and inserted into vector *pGEX4T-2* yielding pGEX4T2-UGT86C4 and pGEX4T2-UGT94F2 respectively. The expression constructs were checked for *in-frame* fusion by DNA sequencing. The gene constructs were transformed into competent *BL21* cells and their expressions were induced by the addition of 0.2 mM IPTG at approximately OD_600_ = ∼0.5. This resulted in the appearance of a new fusion polypeptide with an expected molecular mass of approximately 68 kDa when resolved on SDS-PAGE. Time-course study revealed that the optimum expression of UGT86C4 as examined from SDS-PAGE profile was obtained after 6 h of induction at 37°C (Figure S9-A). UGT94F2 was expressed after 2 h of IPTG induction at 37°C (Figure S9-B).

## Conclusion

Understanding of various enzymatic or regulatory steps involved in secondary metabolite biosynthesis is a prerequisite for metabolic engineering. Despite numerous studies on the occurrence, chemical structure, and varying pharmaceutical activity of picrosides, their biosynthesis remains to be poorly understood. Pathway intensification leading to enhanced production of metabolites in host plant or heterologous production in microbial systems can prove to be important for their scalable production. Glucosylation of iridoids is thought to be one of the key steps in the biosynthesis of picrosides. However, no gene encoding glucosyltransferase responsible for picroside synthesis has been isolated. In this study, two putative UDP-glucosyltransferases, UGT86C4 and UGT94F2 were isolated from *P. kurrooa* using a rapid amplification of cDNA ends method. Using molecular modeling and structural analysis, we were able to show that UGT86C4 is a flavonoid- specific UDP-glucosyltransferase having maximum binding efficiency for kaempferol, whereas UGT94F2 was predicted to be an iridoid-specific UDP-glucosyltransferase having maximum binding affinity for 7-deoxyloganetin. Amino acid analysis suggested that UDP-glucose is the donor substrate in both the UGTs. This study also establishes the role of these genes in response to different external stimuli. UGT86C4 and UGT94F2 showed varied expression in different tissues of the plant. Rapid increase in the transcript level of these genes, following MeJA, SA or 2, 4-D treatments suggest their role in biotic and abiotic stresses. To have a better understanding into the regulation of these UGTs, promoter regions of both UGT86C4 and UGT94F2 were isolated and the key *cis*-regulatory elements including those involved in MeJA, SA and auxin responsiveness were identified. Moreover, the picroside content in different tissues corroborated well with the gene expression of UGT94F2 and the change in picroside levels also showed a positive relationship with the expression pattern of UGT94F2 with elicitor treatment. The cloning and characterization of UGTs as well as their promoter regions from *P. kurrooa* not only constitutes an important step unravelling the key committed step of the picroside biosynthetic pathway, but also provides a fresh prospect to understand the regulation of the iridoid pathway genes in this high value medicinal plant. For homologous modulation of iridoid biosynthetic pathway, we have developed an efficient *Agrobacterium* mediated transformation system [28] and characterized other key regulatory genes of picrosides pathway viz. geraniol 10-hydroxylase (GenBank ACC. No. HM187585), cytochrome P450 reductase (GenBank ACC. No.JN968968) and phenylalanine-ammonia lyase (GenBank ACC. No. JQ996410). We tend to employ the homologous metabolic engineering to understand the regulatory role of UGTs and other identified genes for enhanced production of picrosides, as higher transcript levels of key regulatory genes have a cascading influence on the up-regulation of many pathway genes thus increasing the overall metabolite levels.

## Supporting Information

Figure S1 **Nucleotide sequences of two UGTs from** ***Picrorhiza kurrooa.*** Nucleotide and the deduced amino acid sequence of UGT86C4 (a) and UGT94F2 (b). 5′ UTR and 3′ UTR are underlined. The dash marks the translation termination codon.
(TIF)

Figure S2 **Multiple sequence alignment of deduced amino acid sequences of UGT86C4 and UGT94F2.** Multiple sequence alignment of UGTs identified from *P. kurrooa* (PkUGT86C4; PkUGT94F2) with other plant orthologs using ClustalW2 multiple alignment tool. *Arabidopsis thaliana* (AtUGT72B1), *Medicago tranculata* (MtUGT71G1; MtUGT85H2), *Gardenia jasmonoides* (GjUGT85A24), *Catharunthus roseus* (CrUGT85A23) *and Vitis vinifera* (VvGT1). The highly conserved PSPG box is shaded in dark grey. Catalytically important amino acid residues are also shown.
(TIF)

Figure S3 **Transmembrane domain prediction of UGT86C4 (A) and UGT94F2 (B) using Phobious web server.**
(TIF)

Figure S4 **Prediction of putative glycosylation sites on UGT86C4 (A) and UGT94F2 (B) using NetNGlyc 1.0 server.**
(TIF)

Figure S5 **Structural alignment of template (2vch-a) with the predicted three dimensional structures of UGT86C4 and UGT94F2.** Template (2vch-a) is shown in cyan color and UGT86C4 is shown in purple color while as UGT94F2 is shown in brown color.
(TIF)

Figure S6 **Ramachandran plot of two UGTs.** Ramachandran plot of UGT86C4 (A) and UGT94F2 (B). The plot calculations on the 3D models of *Picrorhiza* UGT proteins were computed with the PROCHECK server. Most favoured regions are coloured red (A, B, L), additional allowed (a, b, l, p), generously allowed ([∼a, ∼b, ∼l, ∼p), and disallowed regions are indicated as yellow, light yellow and white regions, respectively.
(TIF)

Figure S7 **Secondary structure analysis of UGT86C4 and UGT94F2 by linear depiction of UGT86C4 (A) and UGT94F2 (B) secondary structure.**
(TIF)

Figure S8 **Chemical structures of flavonoid and iridoid aglycones used as the glucosyl acceptors to examine the substrate specificity of UGT86C4 and UGT94F2 using molecular modeling and docking analysis.**
(TIF)

Figure S9 **Time-course heterologous expression of UGT86C4 (A) and UGT94F2 (B) in** ***E. coli*** **.** Total protein extracts of *E. coli* cells were used for detection by SDS-PAGE and stained with coomassie blue. Lane M, Protein Molecular Weight Marker. Lane C, induction of control (vector only), expression of recombinant protein after 0 h (U), 2 h, 4 h and 6 h after induction with 0.2 mM IPTG at 37°C.
(TIF)

Table S1 **List of primers used in the study.**
(DOCX)
