# Supplementary figures and images for: Molecular Characterization of UGT94F2 and UGT86C4, Two Glycosyltransferases from Picrorhiza kurrooa: Comparative Structural Insight and Evaluation of Substrate Recognition
Source: PLoS One. 2013 Sep 16;8(9):e73804. doi: 10.1371/journal.pone.0073804 (PMC3774767; doi:10.1371/journal.pone.0073804)

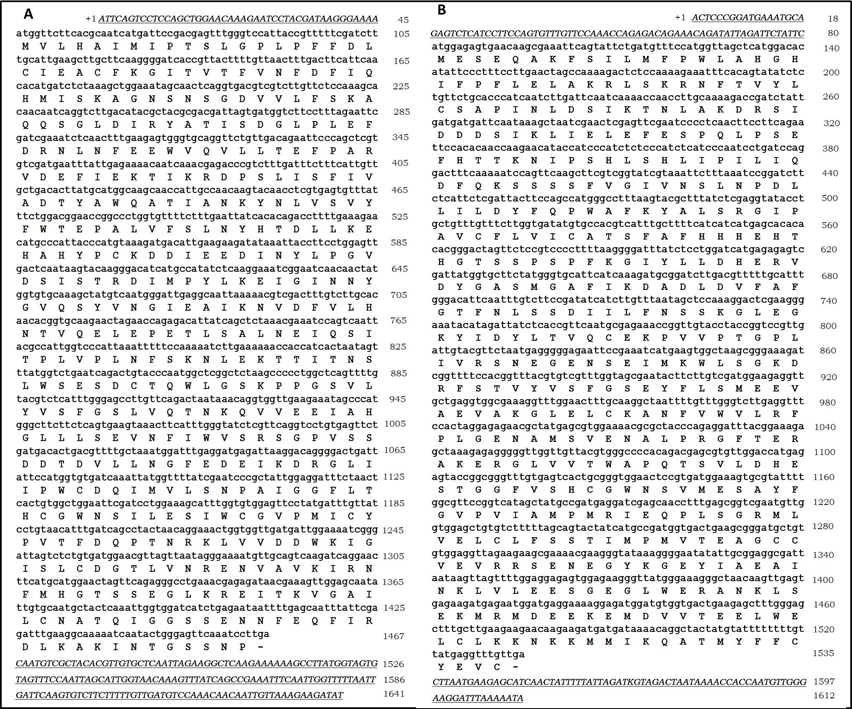

Supplement: Figure S1 — Nucleotide sequences of two UGTs from Picrorhiza kurrooa. Nucleotide and the deduced amino acid sequence of UGT86C4 (a) and UGT94F2 (b). 5′ UTR and 3′ UTR are underlined. The dash marks the translation termination codon. (TIF) [file pone.0073804.s001.tif]

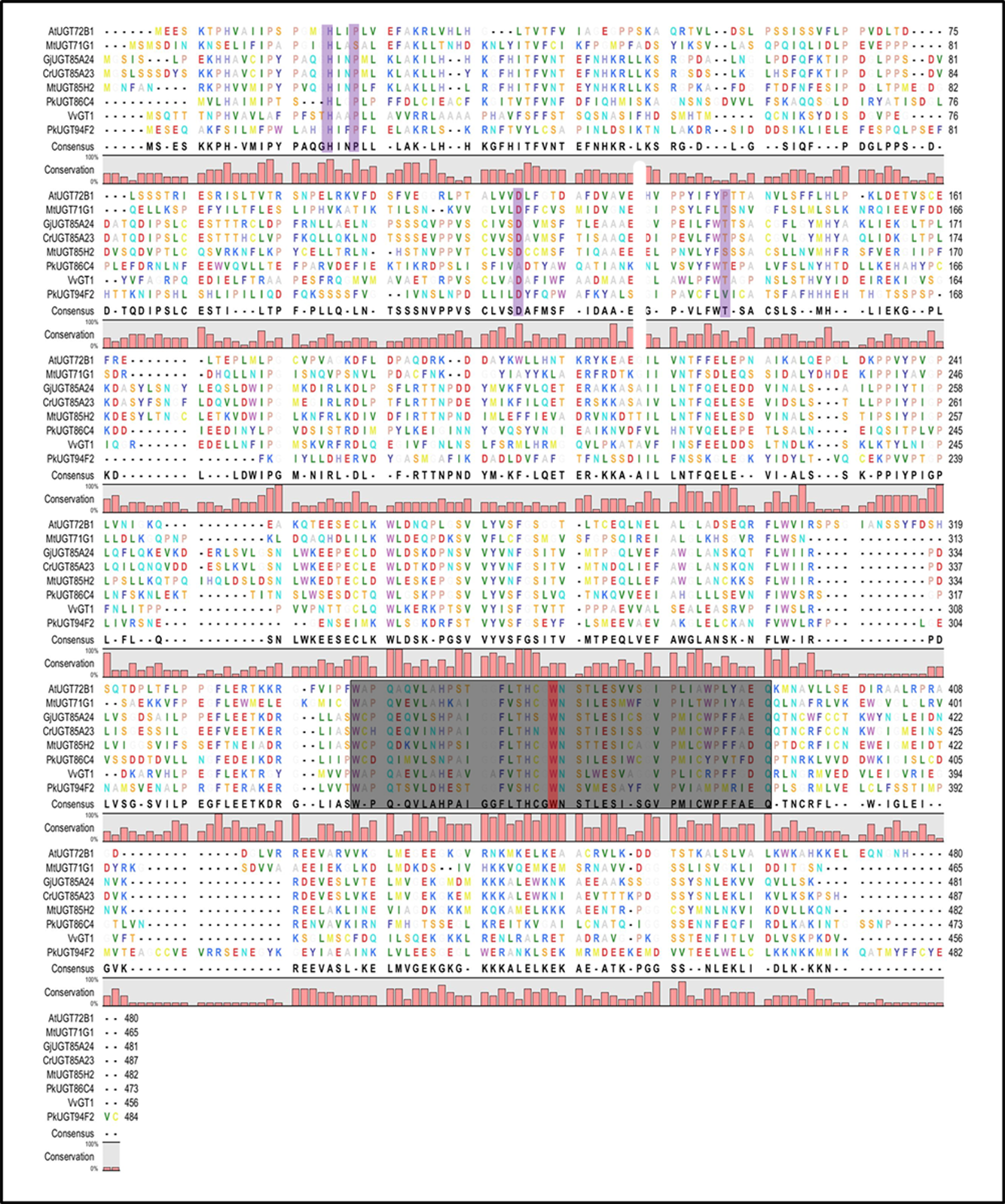

Supplement: Figure S2 — Multiple sequence alignment of deduced amino acid sequences of UGT86C4 and UGT94F2. Multiple sequence alignment of UGTs identified from P. kurrooa (PkUGT86C4; PkUGT94F2) with other plant orthologs using ClustalW2 multiple alignment tool. Arabidopsis thaliana (AtUGT72B1), Medicago tranculata (MtUGT71G1; MtUGT85H2), Gardenia jasmonoides (GjUGT85A24), Catharunthus roseus (CrUGT85A23) and Vitis vinifera (VvGT1). The highly conserved PSPG box is shaded in dark grey. Catalytically important amino acid residues are also shown. (TIF) [file pone.0073804.s002.tif]

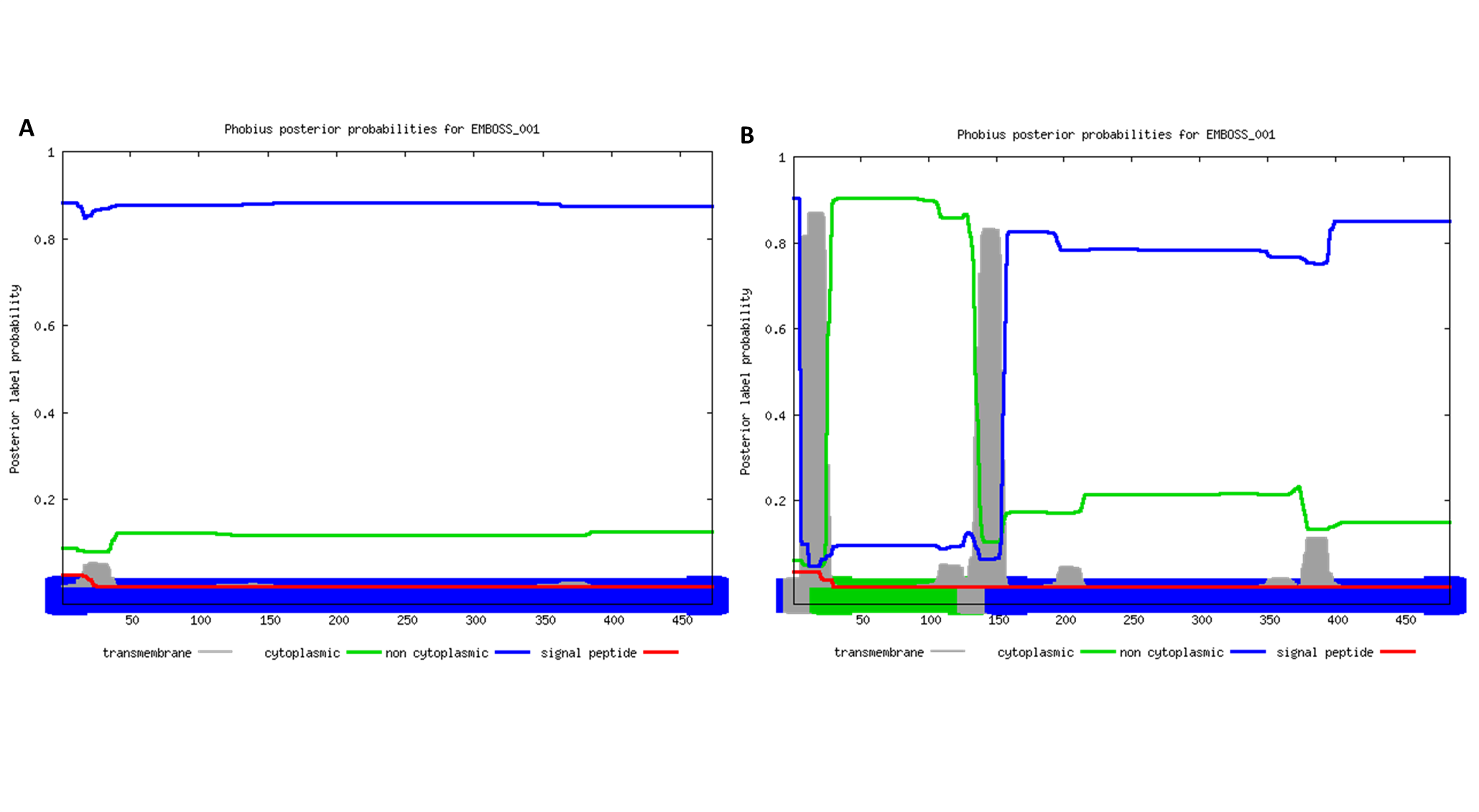

Supplement: Figure S3 — Transmembrane domain prediction of UGT86C4 (A) and UGT94F2 (B) using Phobious web server. (TIF) [file pone.0073804.s003.tif]

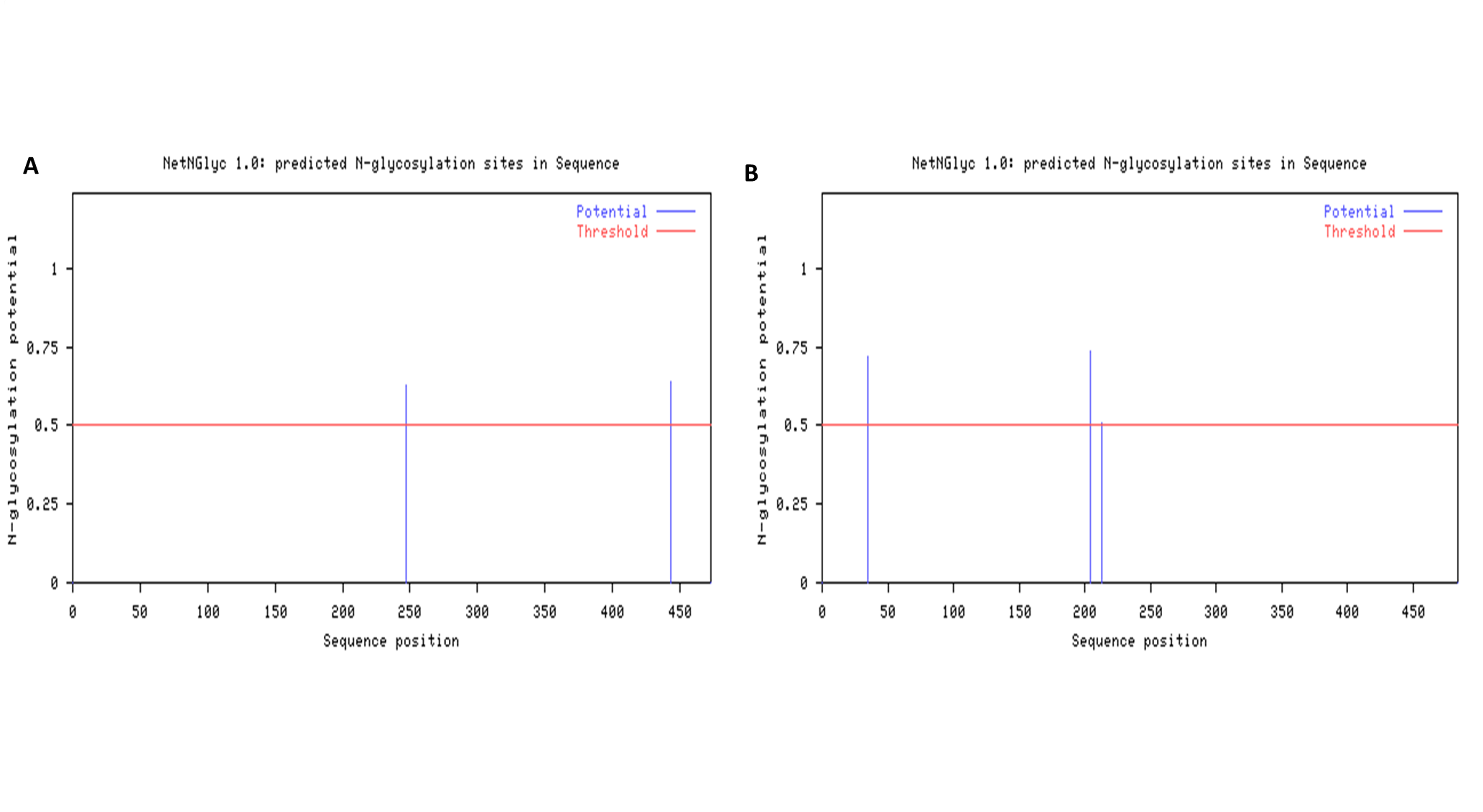

Supplement: Figure S4 — Prediction of putative glycosylation sites on UGT86C4 (A) and UGT94F2 (B) using NetNGlyc 1.0 server. (TIF) [file pone.0073804.s004.tif]

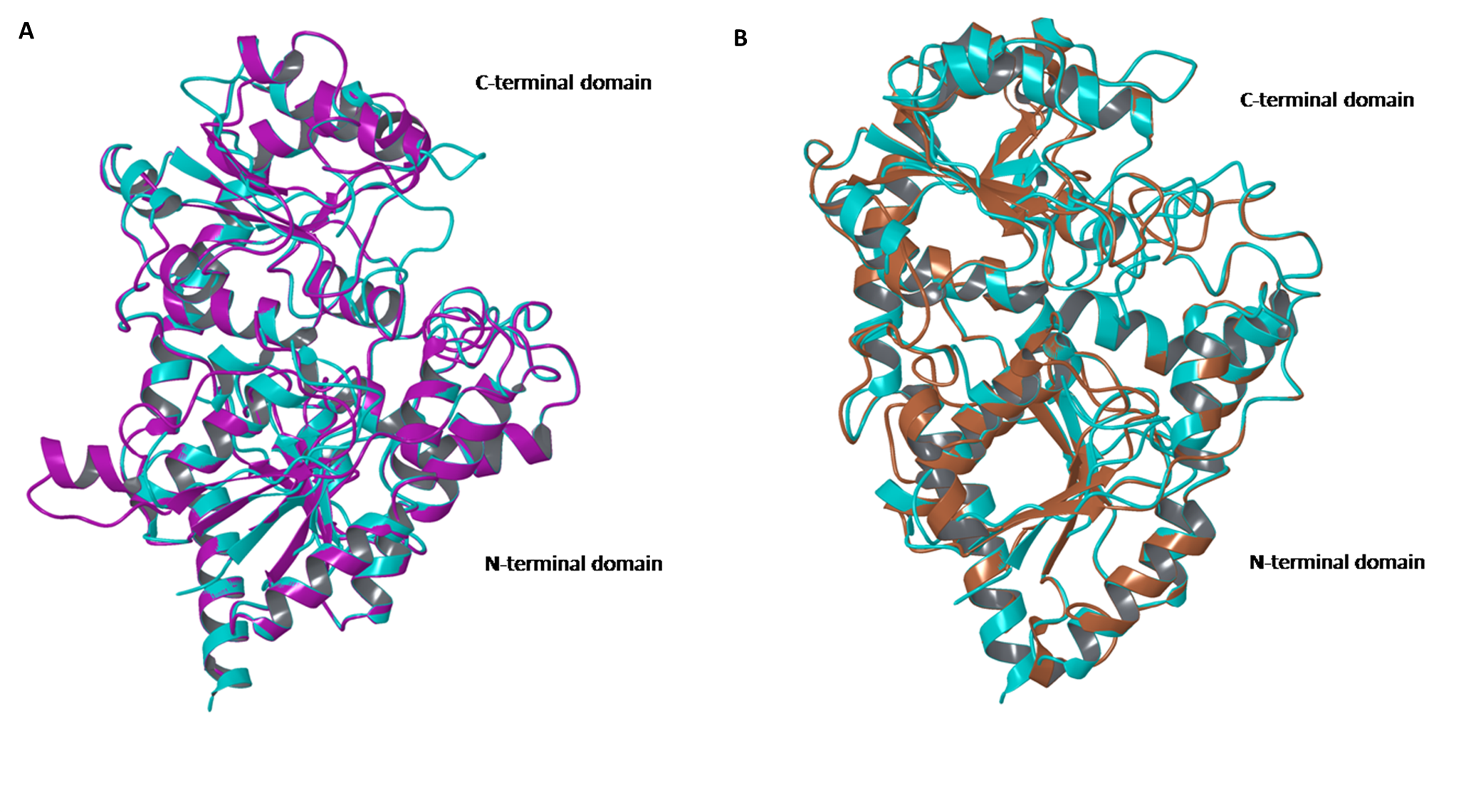

Supplement: Figure S5 — Structural alignment of template (2vch-a) with the predicted three dimensional structures of UGT86C4 and UGT94F2. Template (2vch-a) is shown in cyan color and UGT86C4 is shown in purple color while as UGT94F2 is shown in brown color. (TIF) [file pone.0073804.s005.tif]

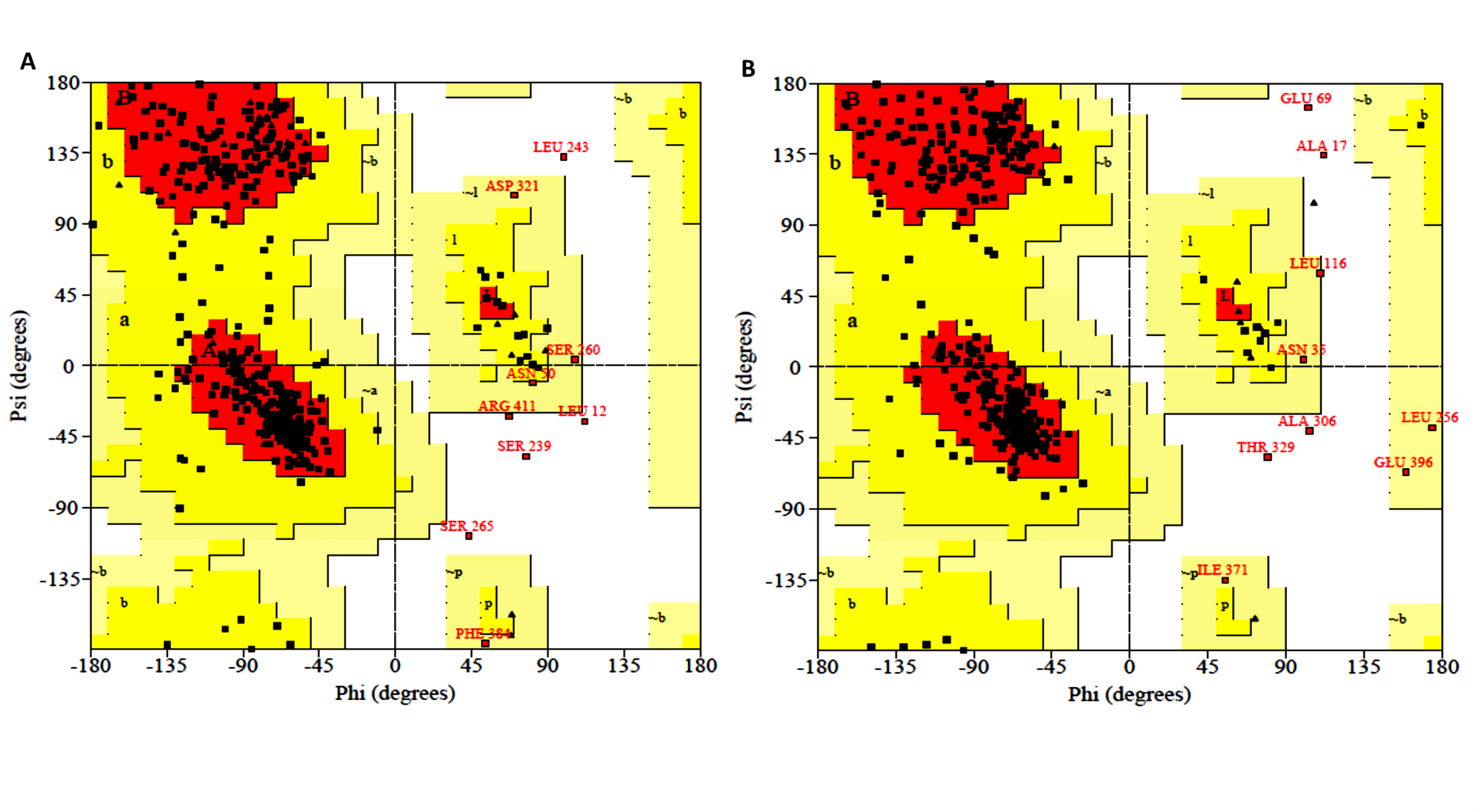

Supplement: Figure S6 — Ramachandran plot of two UGTs. Ramachandran plot of UGT86C4 (A) and UGT94F2 (B). The plot calculations on the 3D models of Picrorhiza UGT proteins were computed with the PROCHECK server. Most favoured regions are coloured red (A, B, L), additional allowed (a, b, l, p), generously allowed ([∼a, ∼b, ∼l, ∼p), and disallowed regions are indicated as yellow, light yellow and white regions, respectively. (TIF) [file pone.0073804.s006.tif]

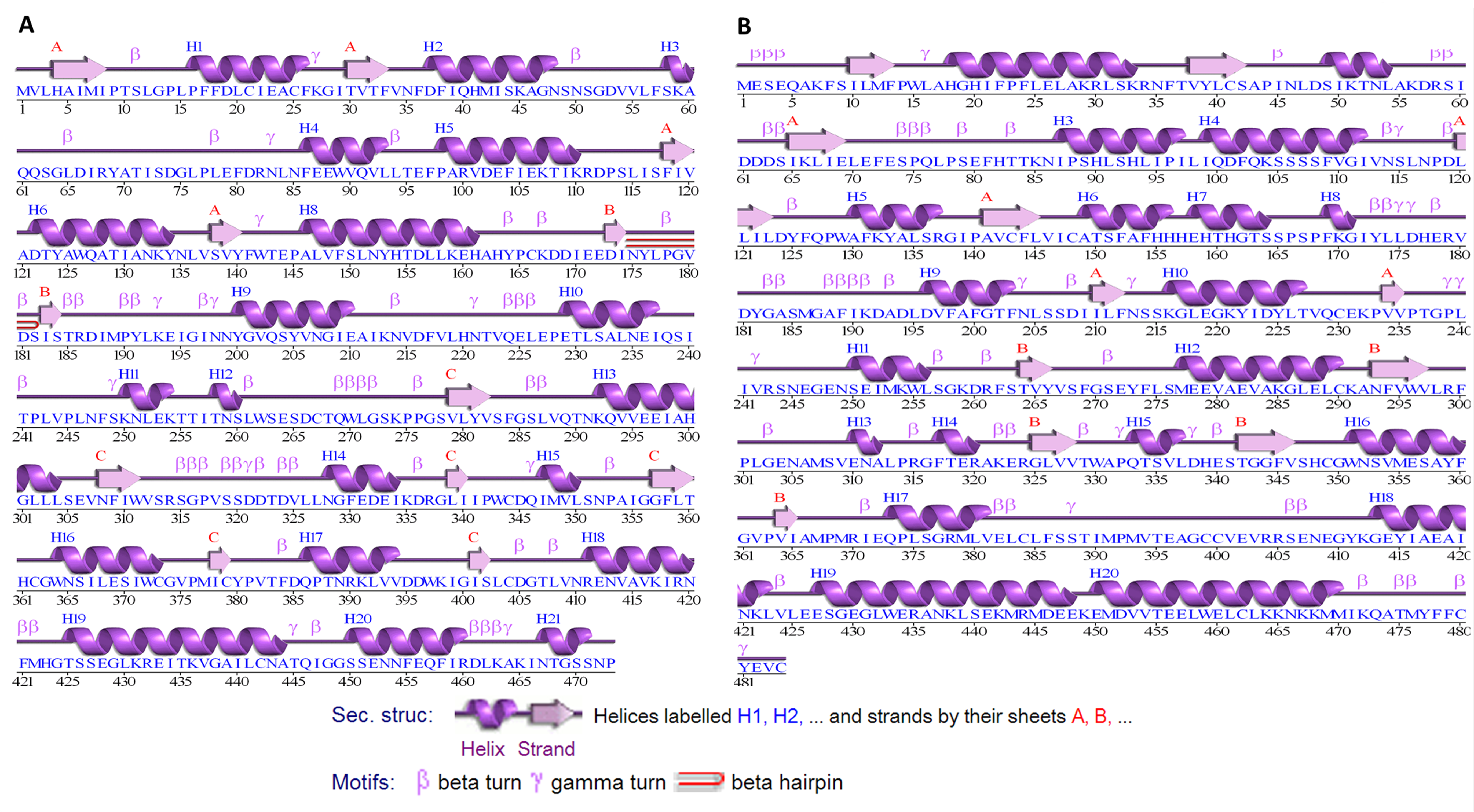

Supplement: Figure S7 — Secondary structure analysis of UGT86C4 and UGT94F2 by linear depiction of UGT86C4 (A) and UGT94F2 (B) secondary structure. (TIF) [file pone.0073804.s007.tif]

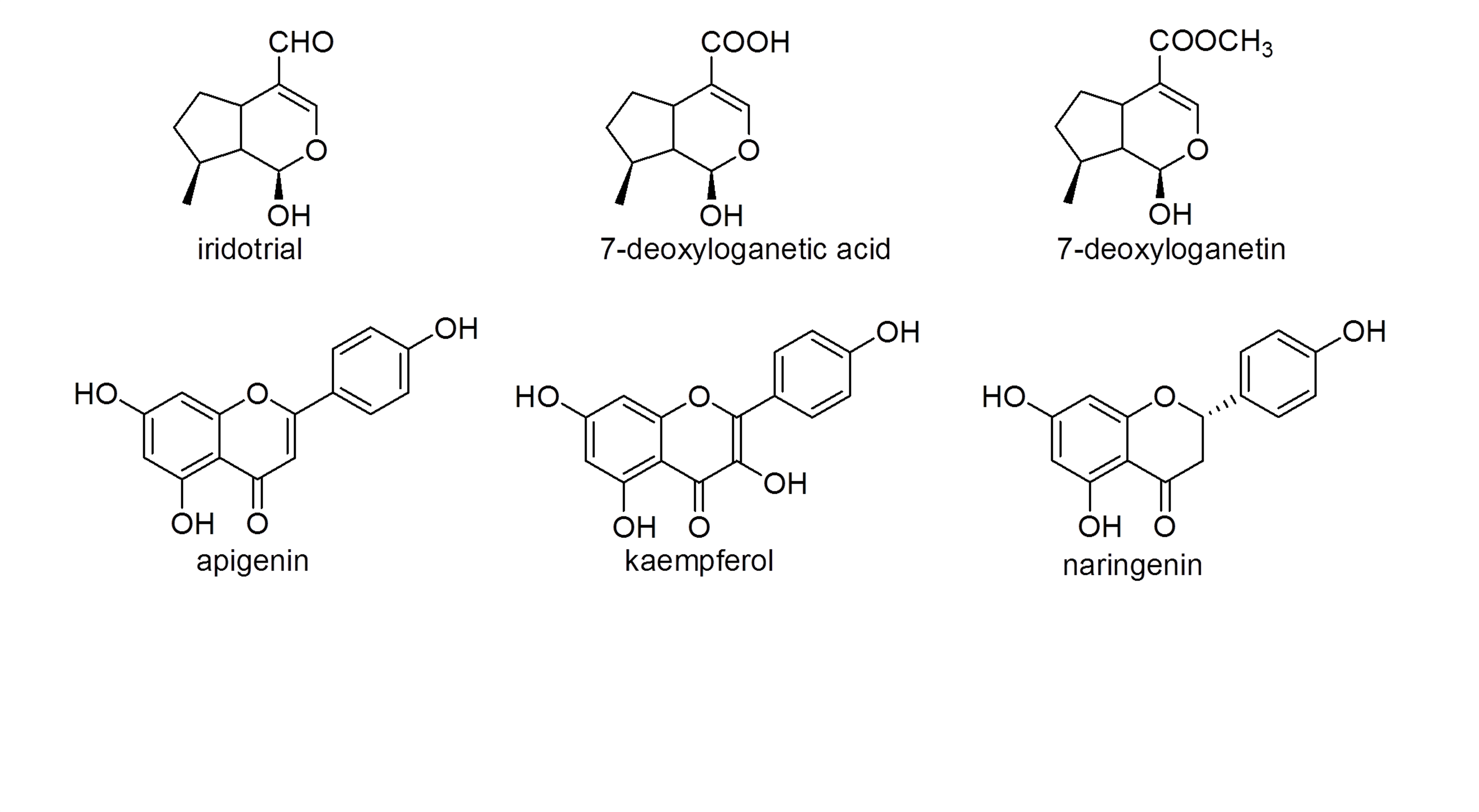

Supplement: Figure S8 — Chemical structures of flavonoid and iridoid aglycones used as the glucosyl acceptors to examine the substrate specificity of UGT86C4 and UGT94F2 using molecular modeling and docking analysis. (TIF) [file pone.0073804.s008.tif]

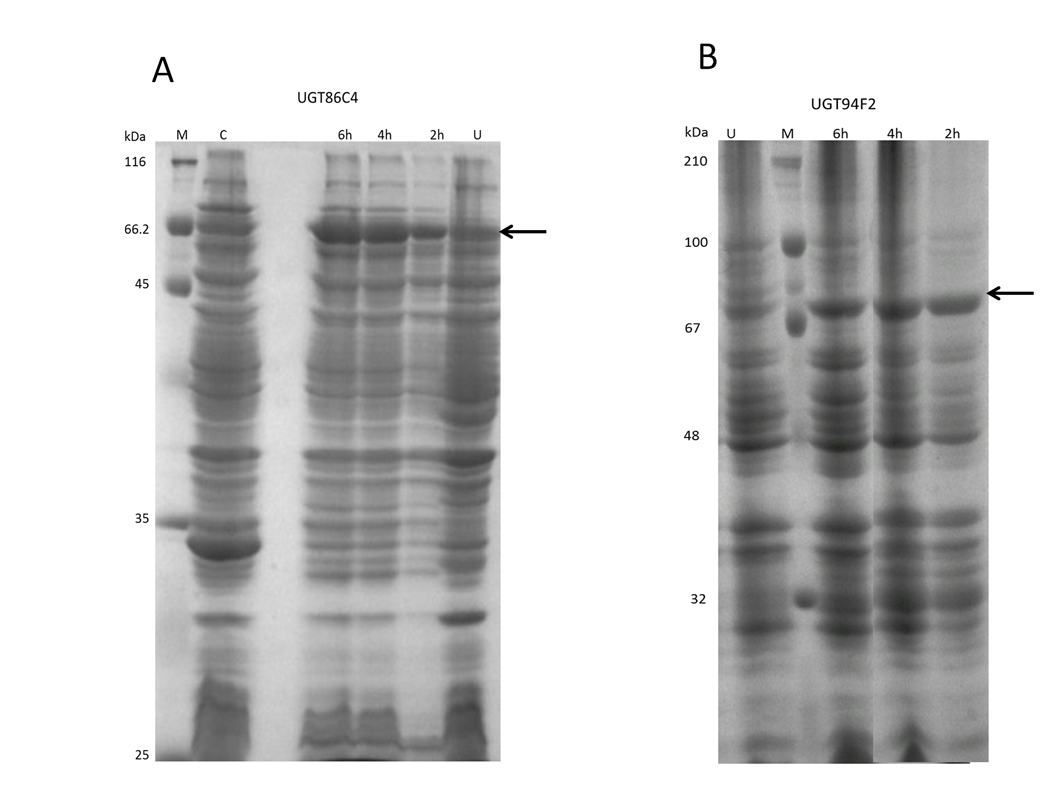

Supplement: Figure S9 — Time-course heterologous expression of UGT86C4 (A) and UGT94F2 (B) in E. coli . Total protein extracts of E. coli cells were used for detection by SDS-PAGE and stained with coomassie blue. Lane M, Protein Molecular Weight Marker. Lane C, induction of control (vector only), expression of recombinant protein after 0 h (U), 2 h, 4 h and 6 h after induction with 0.2 mM IPTG at 37°C. (TIF) [file pone.0073804.s009.tif]
