# Supplementary material for: Molecular Characterization of UGT94F2 and UGT86C4, Two Glycosyltransferases from Picrorhiza kurrooa: Comparative Structural Insight and Evaluation of Substrate Recognition
Source: PLoS One. 2013 Sep 16;8(9):e73804. doi: 10.1371/journal.pone.0073804 (PMC3774767; doi:10.1371/journal.pone.0073804)
Supplement: Table S1 — List of primers used in the study. (DOCX) [file pone.0073804.s010.docx]

**Table S1.** List of primers used in the study

| S. No. | Primer name | Direction | Sequence ( 5´ 3)´ |
| --- | --- | --- | --- |
|  | **Degenerate primers** | |  |
|  | degUGTF1 | Forward | TSNGTNGCNTAYGTNTSNTTYGG |
|  | degUGTF2 | Forward | GTNGCNTAYGTNTSNTTYGG |
|  | **3´ RACE primers** | |  |
|  | Adapter OligodT | Reverse | CTGTGAATGCTGCGACTACGAT_(23)_ |
|  | 3´ RACE Adapter | Reverse | CTGTGAATGCTGCGACTACGAT |
|  | **5´ RACE primers** | |  |
|  | UGT1-5race-out | Reverse | CGA TAC CGA CGA AGC TTG AAC TGG ATT |
|  | UGT1-5race-in | Reverse | CCG GCA ACA CCG ATT CTA AAC TAG ATT |
|  | UGT2-5race-out | Reverse | CTC TTG GCT AGC TCA AGG AAA GGG AAT |
|  | UGT2-5race-in | Reverse | ATT CTA AAC TAG ATT AGC TCA AGG AAA |
|  | UPM* | Forward/ Reverse | Long: 5'-CTAAT ACGAC TCACT ATAGG GCAAG CAGTG GTATC AACGC AGAGT-3'  Short: 5'-CTAAT ACGAC TCACT ATAGG GC-3' |
|  | NUP* | Forward/ Reverse | 5'-AAGCA GTGGT ATCAA CGCAG AGT-3' |
|  | **Full length cloning** | |  |
| 11. | PkUGT1Full-F Forward | | **ATG** GTT CTT CAC GCA ATC ATG A |
| 12. | PkUGT1Full-R Reverse | | **TCA** AGG ATT TGA ACT CCC AGT A |
| 13. | PkUGT2Full-F Forward | | **ATG**GAGAGTGAACAAGCGAAA |
| 14. | PkUGT2Full-R Reverse | | **TCA** ACA AAC CTC ATA ACA AA |
|  | **Full length gene expression** | |  |
|  | BAMUGT1F | Forward | *GGA TCC* **ATG** GTT CTT CAC GCA ATC ATG A |
|  | NOTUGT1R | Reverse | *GCG GCC GC* **TCA** AGG ATT TGA ACT CCC AGT A |
|  | BAMUGT2F | Forward | *GGATCC* **ATG**GAGAGTGAACAAGCGAAA |
|  | NOTUGT2R | Reverse | *GCGGCCGC* **TCA** ACA AAC CTC ATA ACA AA |
|  | **Real-time primers** | |  |
|  | rtUGT1F | Forward | TCTCAAGGAAATCGGAATCA |
|  | rtUGT1R | Reverse | AATTGACTGGATTTCGTTTA |
|  | rtUGT2F | Forward | TTCCACTAGGAGAGAACGCTA |
|  | rtUGT2R | Reverse | ACGCACTTTCCATCACGGAGTT |
|  | rtActinF | Forward | GAGAGTTTTGATGTCCCTGCCATG |
|  | rtActinR | Reverse | CAACGTCGCATTTCATGATGGAGT |
|  | **Promoter primers** | |  |
|  | gwUGT1-out | Reverse | AGATCATGTGTTGAATGAAGTCAAAGTT |
|  | gwUGT1-in | Reverse | GACCCAAACTCGTCGGAATCATGAT |
|  | gwUGT2-out | Reverse | GTGCAGAACAGAGATATACTGTGA |
|  | gwUGT2-in | Reverse | AATATGTGTCCATGAGCTAACCAT |
|  | Walker-AP1* | Forward | GTAAT ACGAC TCACT ATAGG GC-3' |
|  | Walker-AP2* | Forward | ACTAT AGGGC ACGCG TGGT-3' |

* Primers marked with a star were provided with the kit, BamH1 and Not1 sites are italicised, start and stop codon sites are underlined. Primers used in were synthesised by Integrated DNA Technologies (<http://www.idtdna.com/site>)
